# Pinpointing Genomic Regions and Candidate Genes Associated with Seed Oil and Protein Content in Soybean through an Integrative Transcriptomic and QTL Meta-Analysis

**DOI:** 10.3390/cells12010097

**Published:** 2022-12-26

**Authors:** Virender Kumar, Vinod Goyal, Rushil Mandlik, Surbhi Kumawat, Sreeja Sudhakaran, Gunashri Padalkar, Nitika Rana, Rupesh Deshmukh, Joy Roy, Tilak Raj Sharma, Humira Sonah

**Affiliations:** 1 Department of Agriculture Biotechnology, National Agri-Food Biotechnology Institute (NABI), Mohali 140306, India; 2 Regional Centre for Biotechnology, Faridabad 121001, India; 3 Department of Botany and Plant Physiology, CCS Haryana Agriculture University, Hisar 125004, India; 4 Department of Biotechnology, Panjab University, Chandigarh 160014, India; 5 Centre of Digital Agriculture, Plaksha University, Mohali 140306, India; 6 Department of Biotechnology, Academic Block 1, Central University of Haryana, Jant-Pali, Mahendragarh, Haryana 123031, India; 7 Division of Crop Science, Indian Council of Agriculture Research (ICAR), Krishi Bhavan, New Delhi 110001, India

**Keywords:** haplotyping, meta-analysis, nutrition, quantitative trait loci, soybean, transcriptomics

## Abstract

Soybean with enriched nutrients has emerged as a prominent source of edible oil and protein. In the present study, a meta-analysis was performed by integrating quantitative trait loci (QTLs) information, region-specific association and transcriptomic analysis. Analysis of about a thousand QTLs previously identified in soybean helped to pinpoint 14 meta-QTLs for oil and 16 meta-QTLs for protein content. Similarly, region-specific association analysis using whole genome re-sequenced data was performed for the most promising meta-QTL on chromosomes 6 and 20. Only 94 out of 468 genes related to fatty acid and protein metabolic pathways identified within the meta-QTL region were found to be expressed in seeds. Allele mining and haplotyping of these selected genes were performed using whole genome resequencing data. Interestingly, a significant haplotypic association of some genes with oil and protein content was observed, for instance, in the case of *FAD2-1B* gene, an average seed oil content of 20.22% for haplotype 1 compared to 15.52% for haplotype 5 was observed. In addition, the mutation S86F in the *FAD2-1B* gene produces a destabilizing effect of (ΔΔG Stability) −0.31 kcal/mol. Transcriptomic analysis revealed the tissue-specific expression of candidate genes. Based on their higher expression in seed developmental stages, genes such as sugar transporter, fatty acid desaturase (FAD), lipid transporter, major facilitator protein and amino acid transporter can be targeted for functional validation. The approach and information generated in the present study will be helpful in the map-based cloning of regulatory genes, as well as for marker-assisted breeding in soybean.

## 1. Introduction

Soybean has emerged as one of the most important crops globally since it is an excellent source of edible oil and protein [1]. Soybean seeds have about 18–21% oil and 40–45% protein content, and are majorly utilized as food, animal feed, cooking oil, and industrial biofuel [2]. It is a major oilseed crop in the global market, contributing more than 50% to global vegetable oil production [3]. The crude soybean oil contains ~55% linoleic acid (18:2), ~21% oleic acid (18:1), ~12% palmitic acid (16:0), ~9% linolenic acid (18:3), and ~4% stearic acid (18:0) [4,5]. Apart from the production of cooking oil, soybean oil has various industrial applications and it is also used in the production of biodiesel [6]. With the increasing demand for soybeans for food and industrial usage, tailoring the seed composition traits has been a long-standing goal for the soybean research community. Determining the genetic basis of seed composition traits is imperative for developing soybean varieties with improved nutritional quality.

The domestication of soybeans has led to the selection of large seeds, which resulted in less protein and more oil content than the wild soybean *Glycine soja* [7]. The soybean oil and protein are important traits; however, these traits are negatively correlated, which is a major bottleneck for soybean improvement [2,8,9]. These traits also correlate with other seed composition and agronomical traits. A negative correlation has been reported between seed size and protein content [10]. A negative correlation was also reported between protein content and yield [11]. However, both positive and negative correlations of seed oil content with yield have been reported in two different environments [12]. Most of the agronomically important traits in soybean are negatively correlated with either oil or protein content, which necessitates a deeper understanding of the genomic regulation of these traits.

The soybean seed oil and protein content are complex traits governed by several small effect genes and quantitative trait loci (QTLs) [13]. Significant efforts have been employed to understand the genetic regulation of these traits and for the exploration of the resulting QTLs in breeding programs [2]. More than 450 QTLs governing oil and protein content have been identified so far using different bi-parental populations in soybean. These studies have utilized different mapping populations relative to their size, types and generation, different numbers and types of molecular markers, and statistical methods. Most of the QTLs cover the long/varying confidence intervals (CIs), which makes it difficult to define the precise chromosomal region associated with a trait. Most of the previously reported studies have identified major QTLs for oil and protein content on chromosome 20 and minor QTLs on other chromosomes. Several loci governing seed oil and protein content in soybean have been identified using the genome wide association studies (GWAS) approach; however, no specific major gene has been pinpointed so far due to the complex inheritance of these traits [13].

Over the last couple of decades, plenty of information related to the QTLs regulating seed oil and protein content has been generated, with incredible efforts made by the global soybean research community. Such data can serve as a precious resource to perform meta-analysis, which will help to pinpoint the QTL positions and for subsequent candidate gene identification. The information of marker positions, LOD values, R2 values, and methods utilized for QTLs mapping is being used for meta-QTL analysis to further narrow down the confidence interval (CI) for the regions associated with these traits. The meta-analysis studies have been successful in narrowing down the CI of QTLs identified in various crops including maize, rice, wheat, and soybean. Several meta-QTL studies have been reported in soybeans related to canopy [14], plant height [15], seed weight [16], seed yield, amino acid content [17,18], fatty acid [19], oil [20] and protein [21] traits, and successfully identified the associated candidate genes.

Moreover, a significant amount of publicly accessible genomics and transcriptomics data could be used to enhance our comprehension of the complicated genetics underlying diverse agronomic traits. Several databases specific to soybean, which are comprehensive web resources designed to store and integrate omics data, have been developed, such as SoyBase (https://www.soybase.org/, accessed on 1 October 2022), SoyKB (https://soykb.org/, accessed on 1 October 2022) BAR (http://bar.utoronto.ca/, accessed on 1 October 2022) and SoyTD (https://soykb.org/SoyTD/, accessed on 1 October 2022). The whole genome resequencing data available for soybean accessions would be helpful for allele mining and the identification of superior haplotypes for the traits of interest. The omics approaches using available genomic and transcriptomic resources would help in dissecting complex traits, such as oil and protein content in soybean. The identification of allele-specific markers would boost marker-assisted selection-based breeding in soybean.

In this study, the information related to previously identified QTLs for seed oil and protein content along with the genetic locations and QTL effects were used to perform the meta-analysis. The meta-QTL region-specific association analysis was performed to pinpoint the locus governing the seed oil and protein content, followed by candidate gene identification. The allele mining and haplotyping analysis were performed for the identification of superior alleles. The expression profiling of genes in different tissues and seed developmental stages was also performed. The expression profile of the candidate genes was confirmed using a quantitative real-time PCR (qPCR) assay. The findings of this study will be useful for the prioritization of candidate genes to improve the seed composition traits in soybeans.

## 2. Materials and Methods

### 2.1. Compilation, Curation and Meta-Analysis of QTLs Information

The comprehensive information regarding QTLs governing seed oil and protein content along with their chromosomal locations, flanking markers, QTL positions, logarithm of odds (LOD) values, phenotypic variations (R2 values), the confidence of interval (CI), experimental population and mapping methods utilized for the identification of QTLs were obtained from the extensive literature search, as well as SoyBase database (www.soybase.org/ (accessed on 5 April 2022)) (Table S1). The start and end positions of the selected QTLs were retrieved from the consensus genetic and physical map (*Glyma.Wm82.a2.v1*) obtained from the SoyBase database (https://www.soybase.org/ (accessed on 5 April 2022)) [22]. The information of 509 and 460 QTLs, respectively, governing seed oil and protein content was collected. The QTLs lacking the information of nearest flanking markers in the consensus map, LOD values, and R2 values were discarded. Subsequently, the meta-analysis was carried out using the BioMercator software tool [23]. The Akaike information criterion (AIC), Bayesian information criterion (BIC), and the average weight of evidence (AWE) were utilized as selection criteria of the best model for the meta-QTL analysis. The model with the lowest Akaike information criteria (AIC) was selected as the best model for meta-analysis. Significant meta-QTLs were considered only if the projected QTLs were more than ten.

### 2.2. Meta-QTLs Region-Specific Association Analysis

Whole genome resequencing information available for 1511 soybean genotypes was retrieved in the form of Single Nucleotide Polymorphisms (SNPs) data from the SoyBase database. The SNPs were filtered based on the quality, and the markers with > 10% missing data and minor allele frequency (MAF) of less than 5% were excluded from further analysis. The association analysis was performed in the R package Genome Association and Prediction Integrated Tools (GAPIT version 3). The principal component analysis (PCA) and kinship were calculated and used for GWAS analysis to avoid the spurious association due to population stratification. In total, three PCs were used, explaining a combined 26% of the total variation and centered kinship with a maximum of three alleles used. Several models including the mixed linear model (MLM), multiple loci mixed linear model (MLMM), compressed MLM (CMLM), Enriched CMLM (ECMLM), Fixed and Random Model Circulating Probability Unification (FarmCPU), and Bayesian information and linkage-disequilibrium iteratively nested keyway (Blink) implemented in the GAPIT were used for the association analysis within the significant meta-QTL regions. The threshold *p*-value < 1 × 10^−5^ was used for the identification of significant associations. The phenotypic trait information for the seed oil and protein content of soybean genotypes was obtained from GRIN-Global resources (https://www.grin-global.org/ (accessed on 15 May 2022)). The SnpEff was used for the functional annotation of the SNPs and the PROVEAN tool was utilized for amino acid variant effect prediction [24,25].

### 2.3. Identification of Candidate Genes

For candidate gene identification, the physical positions of the nearest flanking markers of identified meta-QTLs were retrieved. The 0.1 Mb upstream and downstream regions flanking the markers were used for the identification of candidate genes. The BioMart tool implemented in the phytozome server (https://phytozome.jgi.doe.gov/pz/portal.html (accessed on 10 May 2022)) was used to retrieve the complete details of the genes within the meta-QTL regions, including gene name, transcript name, start and end positions, PFAM, SMART, KOG ID, and GO attributes. The genes identified within the meta-QTL regions were categorized into three different groups: transcription factors, transporters, lipids, and protein synthesis/metabolic pathways-related genes. The candidate genes related to fatty acids and the protein biosynthesis/accessory pathway with SNPs causing missense and non-sense mutations in their corresponding alleles were prioritized for subsequent studies. The protein tertiary (3D) structure was developed using the PHYRE V2.0 (Protein Homology/analogY Recognition Engine) online tool (http://www.sbg.bio.ic.ac.uk/~phyre2/html/page.cgi?id=index (accessed on 10 June 2022)). The changes in protein stability upon missense mutations were predicted based on changes in Gibbs free energy (ΔΔG), or the difference between the Gibbs free energy ΔG of the mutant structure and the ΔG of the wild-type structure using the DynaMut2 tool [26].

### 2.4. Haplotypic Characterization of Selected Genes

The publicly available whole genome resequencing data for 40× USB (United soybean board) were used for the haplotypic analysis of selected candidate genes using the SNPviz software tool implemented in the SoyKb database (https://soykb.org/SNPViz2/ (accessed on 20 June 2022)). The chromosome number and gene start and end positions were added in the SNPviz tool and a phylogeny tree was constructed using the default Unweighted Pair Group Method with Arithmetic Mean (UPGMA) method. The t-test was performed to check the significance of difference between haplotypes.

### 2.5. Transcriptomics and Co-Expression Network Analysis

The expression analysis of candidate genes within the meta-QTL region was performed across different tissues and environmental conditions. The publicly available raw reads of RNA-Seq data related to seed transcriptomics were retrieved from the National Center for Biotechnology Information-Sequence Read Archive (NCBI-SRA) database in the fastq format (www.ncbi.nlm.nih.gov/sra (accessed on 20 May 2022)) and subsequently analyzed using the CLC Genomics workbench (www.qiagenbioinformatics.com/ (accessed on 30 May 2022)). The raw sequence data in the form of fastq file of bio-projects PRJNA197251, PRJNA140081, and PRJNA388955 were also used for the expression analysis. This dataset represents the different stages of seed development and other tissues. The different seed-specific compartment tissues isolated using laser capture micro-dissection were also used to study the expression of candidate genes. The raw reads were processed based on the quality and other parameters. The processed reads were mapped to the reference genome *Glyma.Wm82.a2.v1*. Based on the reads mapped at each gene locus and normalized expression in terms of Reads per Kilobase of the transcript per Million (RPKM), mapped read values were calculated. The expressions of all genes in different tissues were analyzed by the Molecular Experiment Viewer 4.9.0 (MEV 4.9.0) tool in the form of a heatmap using the hierarchical clustering method based on Pearson correlation (http://www.tm4.org/mev.html (accessed on 5 June 2022)). The expressions of selected genes in different tissues and seed development stages were also studied using the 4085 RNA-seq libraries of soybean implemented in PPRD (http://ipf.sustech.edu.cn/pub/ (accessed on 10 June 2022)).

### 2.6. Quantitative Real Time PCR

The soybean plants were grown up to maturity under controlled environmental conditions. The leaf, stem, root, and matured seed tissue were collected for total RNA isolation. The total RNA isolation was carried out using the TRIZOL method. The quality and quantity of RNA were checked by agarose gel electrophoresis and a nanodrop spectrophotometer. The cDNA synthesis was carried out from isolated RNA using a PrimeScript™ 1st strand cDNA Synthesis Kit as per the manufacturer’s protocol. The synthesized cDNA was stored at −20 °C until further use. A quantitative real-time PCR was performed for six genes on the Bio-Rad CFX connect machine using Bio-Rads iTaq Universal SYBR Green Supermix. Each 10 μL reaction consisted of 5 μL of SYBR Green Supermix, 2 μL of cDNA, 0.5 μL of each primer, and 2 μL of dH_2_O. All the experiments were conducted with three biological and three technical replicates. The qPCR primers were designed using QuantPrime (Table S2). The elongation factor (ELF1) was used as the internal control gene. The reaction setup was as follows: initial denaturation at 95 °C for 1 min followed by 40 cycles of denaturation at 95 °C for 10 s, annealing for 20 s at 55 °C, and extension for 20 s at 72 °C in 96-well optical reaction plates. In the end a stepwise increase in temperature was performed from 65 °C to 95 °C where an increment of 0.5 °C was undertaken every 5 s. The 2^−(∆∆Ct)^ method was used for data analysis and the relative expression data were obtained.

## 3. Results

### 3.1. Meta-QTLs Governing Seed Oil and Protein Content in Soybean

**Figure 1:**
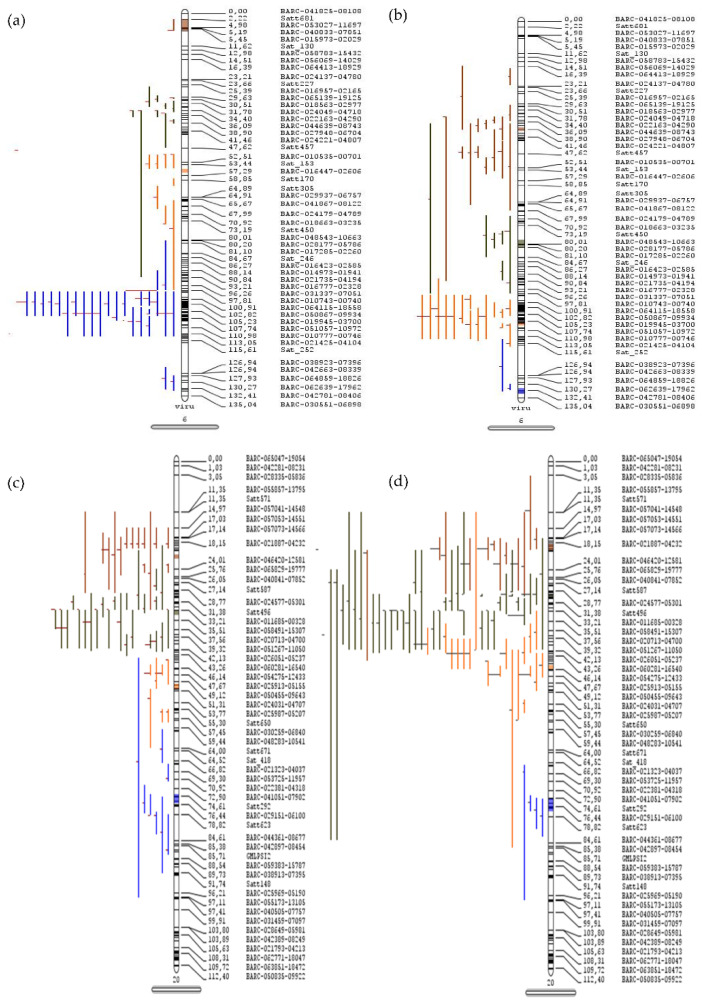
Meta-quantitative trait loci for seed oil and protein content in soybean. The QTLs projected on chromosome 6 for (**a**) seed oil and (**b**) protein content and on chromosome 20 for (**c**) seed oil and (**d**) protein content. The vertical bars with rounded ends represent the chromosome, the colored lines on left side represent the QTLs, and the right sides of the bars indicate the position (cm) and name of marker.

The detailed statistics of 509 and 460 QTLs respectively governing seed oil and protein content were used for the meta-QTL analysis. A maximum of 145 QTLs were localized on chromosome 20, followed by 81 QTLs on chromosome 6 of *Glycine max*. The meta-QTL was defined if the projected number of QTLs was more than three and a total of 55 and 58 meta-QTLs for oil and protein content, respectively, were identified. A total of 14 and 16 significant meta-QTLs having more than ten QTLs were identified for seed oil and protein content, respectively, in soybean (Table 1). A total of 22 meta-QTLs regions related to the oil and protein content were found to be overlapping, while some of the other meta-QTLs were present within one Mb region apart. Four major meta-QTLs were present on chromosome 20, with the overlapping region for seed oil and protein content (Table 1 and Table S3). The maximum numbers of projected QTLs within the defined meta-QTL for oil content were present on chromosomes 6 (MetaQTL-OC_6.1) and 20 (MetaQTL-OC_20.2), wherein each has 26 projected QTLs, and the MetaQTL-PC_20.2 for protein content has 37 projected QTLs (Figure 1, Figures S1 and S2). The MetaQTL-OC_20.2 (29.63–30.01 cm) and MetaQTL-PC_20.2 (29.82–30.06 cm), and all other meta-QTLs present on chromosome 20, are common for the seed oil and protein content.

### 3.2. Region Specific Association Analysis

**Figure 2:**
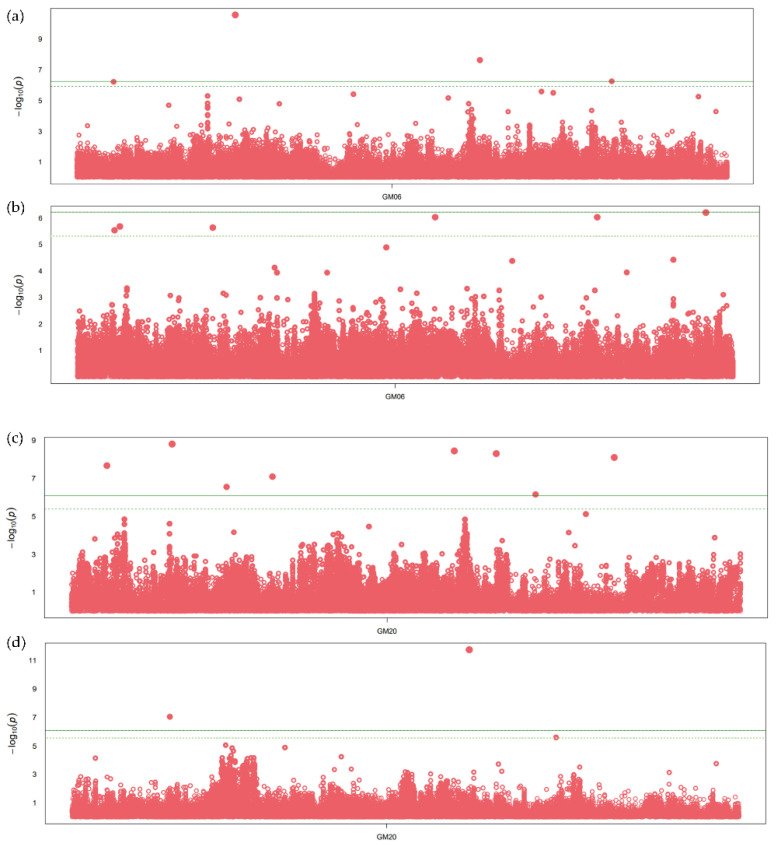
Manhattan plot showing significant SNP peaks and candidate genes on chromosome 6 associated with (**a**) seed oil content and (**b**) protein content, and chromosome 20 associated with (**c**) seed oil and (**d**) protein content. The candidate genes are shown in the vicinity of significant SNP peaks. The green line represents the threshold level based on Bonferroni correction.

The SNPs within the two meta-QTLs with large numbers of projected QTLs and overlapping regions for oil and protein content were further considered for the association analysis. The SNPs with significant association, having *p*-values less than 10 e^−6^, were observed on chromosome 6 and 20 (Figure 2). A total of 12 and seven SNPs were found significantly associated with seed oil and protein content, respectively, on chromosome 6 (Table S4). The SNPs on chromosome 6 are significantly associated with oil and protein content across all GWAS methods. A total of three genes, namely, *Glyma.06G254700* (fatty acid metabolism), *Glyma.06G254400* (*Myb* transcription factor), and *Glyma.06G254900* (*Diacylglycerol kinase*), were identified in the flanking region of significantly associated SNPs (Figure 2a), along with two genes related to amino acid, namely, *Glyma.06G253600* (methionine degradation), *Glyma.06G253700* (organic solute transporter) (Figure 2b). Similarly, nine and five SNPs were associated with seed oil and protein content, respectively, on chromosome 20 (Figure 2). The SNPs on chromosome 20 show a significant association for oil content in all GWAS methods, with the *p*-values ranging from 7.95 × 10^−11^ to 1.32 × 10^−6^ (Table S4). Five genes, namely, *Glyma.20G108800* (mitochondrial pyruvate carrier), *Glyma.20G111000* (*FAD2-1B*, omega-6 fatty acid desaturase), *Glyma.20G125600* (ABC transporter), *Glyma.20G127800*, and *Glyma.20G129500* (triacylglycerol degradation) (Figure 2c), and two genes (*Glyma.20G103200* and *Glyma.20G129400*) related to amino acid biosynthesis were identified in the flanking region of an SNP significantly associated with protein content (Figure 2d). In the flanking regions of significant SNPs, the other important genes related to glycolysis, transporter and transcription factors were also identified.

### 3.3. Candidate Genes for Seed Oil and Protein Content in Soybean

For the candidate genes identification, meta-QTL regions along with a 0.1 Mb flanking region was considered. A total of 9122 genes belonging to diverse functional groups were identified within all the meta-QTL regions (Table S5). Most of the identified genes were transporters, transcription factors, and genes involved in fatty acid and protein biosynthetic pathways. An average of 81 genes were present within the significant meta-QTL regions. A minimum of 10 genes were identified in the MetaQTL-OC_02.4, MetaQTL-OC_06.2, and MetaQTL-OC_15.1, and the maximum numbers of 383 genes were present in MetaQTL-OC_16.3 followed by 378 genes in the MetaQTL-OC_18.3 region. About 26 genes related to oil and protein content were identified in close proximity to significantly associated SNPs. The sugar transporter (*Glyma.20G066500*), ABC transporter (*Glyma.20G066600*), Myb (*Glyma.20G068700*), AP2 (*Glyma.20G070100*), Acyltransferase (*Glyma.20G070400*), Sucrose-phosphate phosphatase (*Glyma.20G070500*), acyl-carrier protein (*Glyma.20G074100*), Far1 (*Glyma.20G076100*), and acetyl-CoA C-acyltransferase (*Glyma.20G077000*) genes involved in lipids and sucrose metabolism were identified in both overlapping MetaQTL-OC_20.2 and MetaQTL-PC_20.2. Similarly, the overlapping MetaQTL-OC_20.3 and MetaQTL-PC_20.3 have several candidate genes, such as TAG biosynthesis, lipid degradation, amino acid transporters, ABC transporter, sugar transporter, L-tryptophan biosynthesis, L-serine and glycine biosynthesis, and many others involved in lipid, protein, and other important pathways. A total of 468 genes were found to be linked to the fatty acid and amino acid pathways, including some transporters based on functional annotation (Table S6). About 112 genes related to the protein metabolism were identified on all the meta-QTLs, and out of them, 98 genes were involved in the amino acid biosynthetic pathway. Three genes related to valine were identified on MetaQTL-PC_17.3, and three genes related to cysteine, glutamine, and methionine synthesis were found on MetaQTL-PC_20.3.

### 3.4. Genotype Variation and Haplotypic Characterization of Candidate Genes

To analyze the genotypic variations, SNPs within the 468 selected genes were identified in 1511 soybean accession. A total of 438 non-synonymous mutations and 57 stop gain (nonsense) mutations were identified (Table S7). Based on the provean score, 57 deleterious mutations were observed in 45 candidate genes (Table S7). A maximum of 25 non-synonymous and four nonsense mutations were identified in the *Glyma.16G128100* gene, followed by 21 non-synonymous mutations in *Glyma.05G214000*. These SNPs have deleterious effects on protein function (Table S7). The *Glyma.20G125600* gene, which is an ABC transporter, has a stop codon at the 96th position of the amino acid sequence.

The acyl-CoA oxidase (*Glyma.03G056400)* involved in fatty acid beta-oxidation has 12 mutations (11 non-synonymous and one nonsense). The genes related to amino acid biosynthesis L-glutamine (*Glyma.19G130800*) have a maximum of 13 mutations (11 non-synonymous and two nonsense), followed by 11 mutations (10 non-synonymous and 1 nonsense) in *Glyma.06G169700* involved in L-isoleucine and L-valine biosynthesis. In the case of transporter genes, a maximum of six mutations (five non-synonymous and one nonsense) were identified in the sugar transporter (*Glyma.08G183500*). The genes involved in sugar biosynthesis, fatty acid metabolism and lipoxygenase contain 5 to 12 mutations, and a maximum of 3 nonsense mutations were found in the sugar biosynthetic gene (*Glyma.05G163600*).

**Figure 3:**
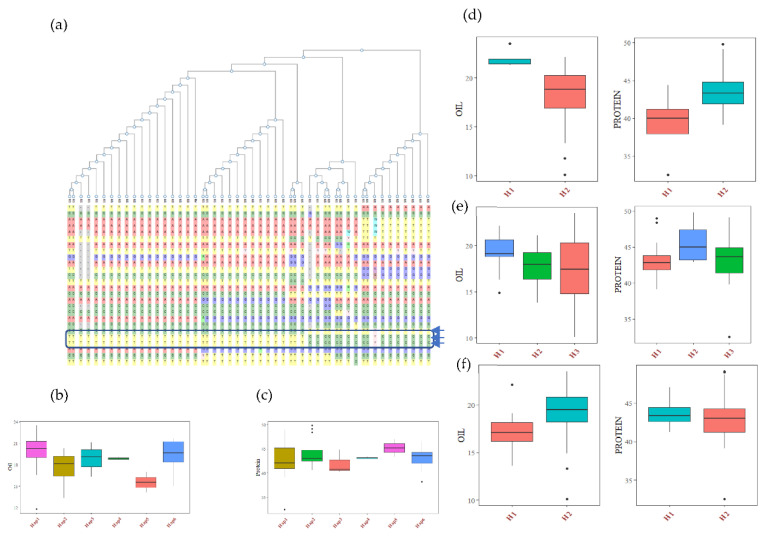
Haplotypic analysis of the (**a**) *FAD2-1B* (*Glyma.20g111000)* gene, showing six haplotypic blocks. (**b**) Haplotypic association of *FAD2-1B* with seed oil, and (**c**) haplotypic association of *FAD2-1B* with seed protein. Haplotypic associations of (**d**), *Glyma.13G035200* (**e**) *Glyma.07G034800* and (**f**) *Glyma.15G049200* with soybean seed oil and protein content.

The haplotype analysis was performed using SNP data of USB lines available within the SNPviz tool of the SoyKB database [27]. A total of 26 SNPs and six haplotypes were observed for the *Glyma.20g111000* (*FAD2-1B*) gene, including 3 non-synonymous SNPs, which clearly distinguished the high oil haplotype (Hap1) group from the low oil haplotype (Hap5) group (Figure 3, Table S8). The average seed oil content of the Hap1 group was 20.22%, and Hap 2 (having one non-synonymous mutation) has a 17.90% seed oil content. Hap 4 and Hap 5 (two non-synonymous mutations) have average oil contents of 18.87% and 15.52%, respectively. The combined AG and CG allele mutation has more drastic effects on oil content. The haplotypes Hap1, Hap2, Hap3, Hap4 and Hap6 had lower average protein contents than that of Hap 5, with a 45.2% average protein content (Figure 3). The haplotype analysis of three other genes based on non-synonymous mutation revealed a significant association with oil and protein (Table S8). The *alcohol dehydrogenase* gene (*Glyma.13G035200*) has one non-synonymous SNP (C to A), which leads to an amino acid change from glycine to aspartic acid at the 79^th^ position of the protein sequence. This mutation causes a 20.6% increase in oil content in the mutant type haplotype compared to the wild type, with 10.2% decreased protein content. This gene is involved in sucrose degradation and the synthesis of acetyl CoA, which is the primary raw material for fatty acid biosynthesis. The *lipoxygenase* gene (*Glyma.07G034800*) has two non-synonymous SNPs, which caused the amino acid change from tyrosine to histidine at the 240th position, and serine to proline at the 257th position of the protein sequence. There were three haplotypes in the *Glyma.07G034800* gene, with H1 having 11% increased oil content over the wild haplotypes, and the H2 haplotype having 5% higher protein content. Similarly, the *Glyma.15G049200* (sugar transporter) gene has one non-synonymous SNP, which causes an amino acid change from arginine to serine at the 246th position of the protein sequence, with the mutant type haplotype having around 8% less oil content (without significant changes in protein content) compared to the wild-type haplotypes. The mutation in the sugar transporter only affects the oil content.

**Figure 4:**
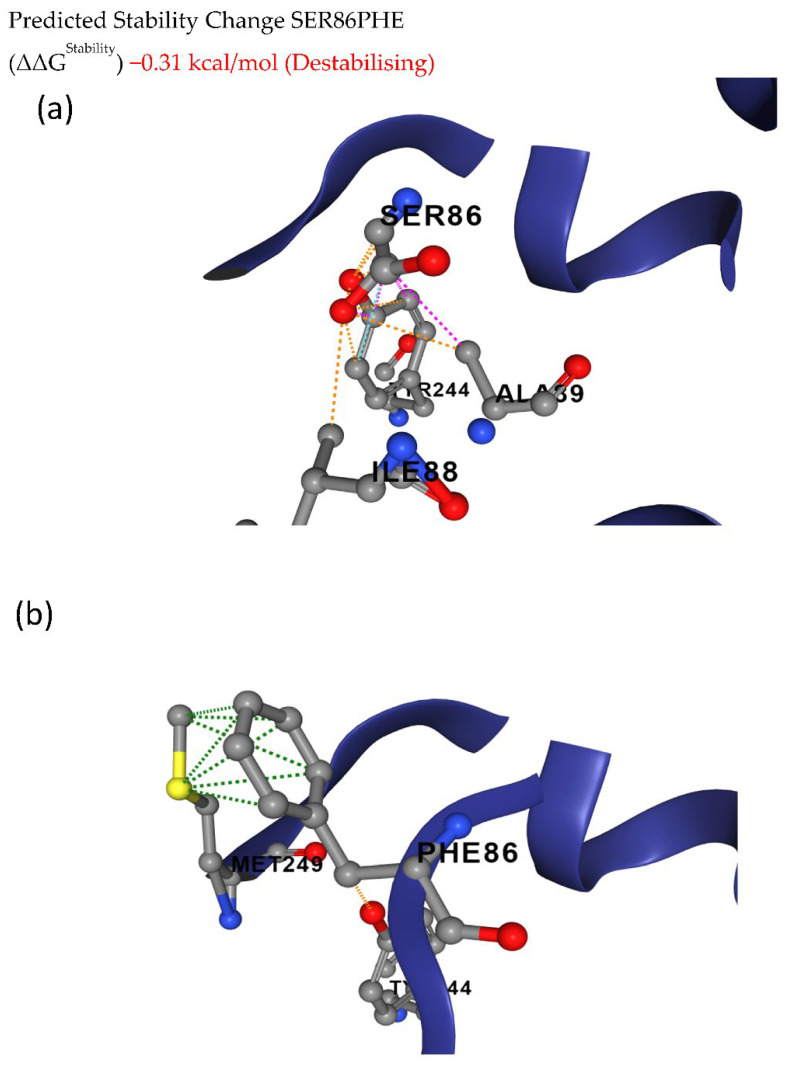
Conformational stability of (**a**) wild and (**b**) mutant FAD2-1B proteins. The deleterious mutation leads to an amino acid change from serine to phenylalanine at the 86th position of the FAD2-1B protein, causing destabilization of the protein structure.

The deleterious mutations were further analyzed to predict the effects of these mutations on structural flexibility. The deleterious mutations leading to S86F in Glyma.20g111000 (FAD2-1B) showed ΔΔG of −0.31 kcal/mol between wild and mutated protein structures, indicating the destabilization of the protein structure (Figure 4).

### 3.5. Transcriptomics Analysis

**Figure 5:**
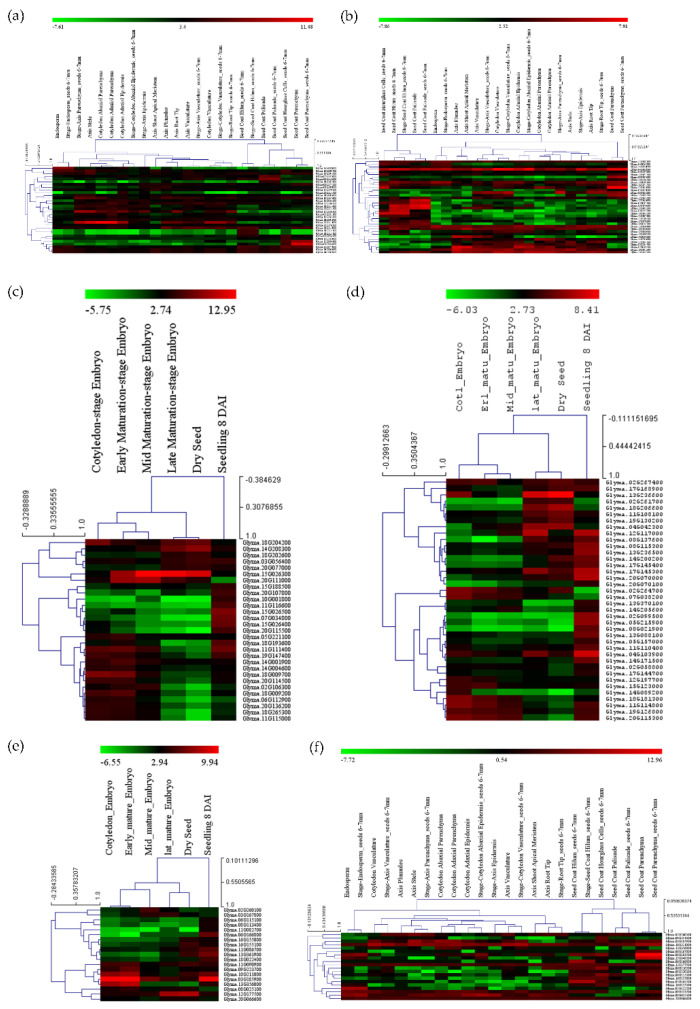
Expressions of genes in different tissues and developmental stages present within the meta-QTL region, (**a**,**c**) genes related to seed oil and protein metabolism/synthesis, (**b**,**d**) the transcription factors, (**e**,**f**) transporter genes.

**Figure 6:**
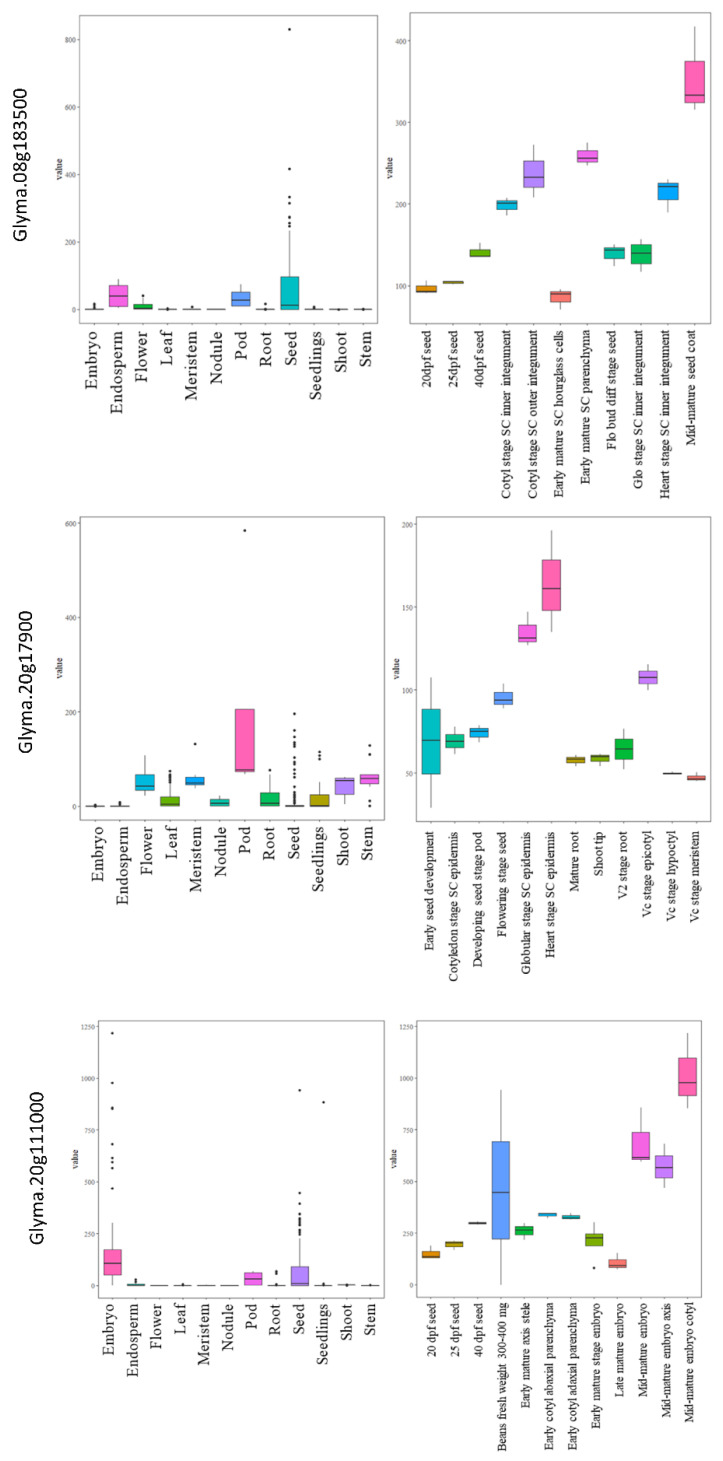
Expressions of three candidate genes related to the seed oil and protein content in different tissues and development stages in soybean.

The expressions for all the selected genes in different tissues of developing seeds were calculated in terms of RPKM values and represented in the form of heatmaps (Figure 5a–f). The three genes highly expressed in the soybean seeds present in MetaQTL-OC_8.3, MetaQTL-PC_20.1 and MetaQTL-OC_20.3 belong to sugar transporter (*Glyma.08G183500*), lipid transporter (*Glyma.20G017900*) and fatty acid desaturase FAD2-1B (*Glyma.20G111000*), respectively (Figure 5 and Figure 6). Sugar is mainly used as a raw material for lipid/protein synthesis and transported from nearby tissues. The sugar transporter (*Glyma.08G183500*) is dominantly expressed in the mid-mature embryo seed coat, and specifically in the seed coat parenchyma cell, from where it transports the sugar. This gene also contains one nonsense and five non-synonymous mutations. The lipid transporter gene (*Glyma.20G017900*) is highly expressed in pod tissue, and the embryo is involved in transporting lipids to developing seeds (Figure 6). The fatty acid desaturase (*Glyma.20G111000*) has seed-specific expression, and is predominantly expressed in the mid-maturation embryo development stage and seed coat parenchyma cells. The MetaQTL-OC_8.2 possesses an acyl-activating enzyme (*Glyma.08G113600*), which shows a high expression in seed coat palisade cells. The two chloroplast genes, namely, *Glyma.11g111100* and *Glyma.11g111400,* are fructose-bisphosphate aldolase-I, which converts the fructose-1,6-bisphosphate to glyceraldehyde 3-phosphate and the dihydroxyacetone phosphate present in MetaQTL-OC_11.3. The *Glyma.11g111400* was observed to be highly expressed in the early mature to mid-mature embryo compared to the late mature embryo and dry seed. This enzyme is involved in glycolysis, which produces the raw material, acetyl CoA, for fatty acid biosynthesis. The lipoxygenase (*Glyma.15g026300*) that catalyzes the oxidation of fatty acids shows very high expression in mid-mature to late mature embryos. The other lipoxygenase (*Glyma.07g034800*) shows specificity to seed coat parenchyma cells.

The transcription factors (bZIP: *Glyma.11G114800*, Myb: *Glyma.18G181300*, bZIP: *Glyma.19G126800*, and AP2: *Glyma.20G115300*) show high expression levels in early mature embryos to mid-mature embryos, whereas AP2: *Glyma.18G206600* has high expression in the late mature embryos and dry seeds. These transcription factors have shown ubiquitous expression in different seed cell compartment expression analyses, except *Glyma.11G114800,* which has high expression in seed parenchyma cells. In the expression analysis, six transcription factors showed predominant expression in palisade and hilum cells (Figure 5).

Out of 458 genes, only 94 genes were finally selected based on their higher expression in soybean seeds, among which 31 genes were related to fatty acids, 14 were related to amino acids and 9 genes were related to sugar (Table S9). The rest of the genes were transcription factors and transporters viz lipid, sugar, and amino acid transporters (Table S9).

### 3.6. QPCR Analysis

**Figure 7:**
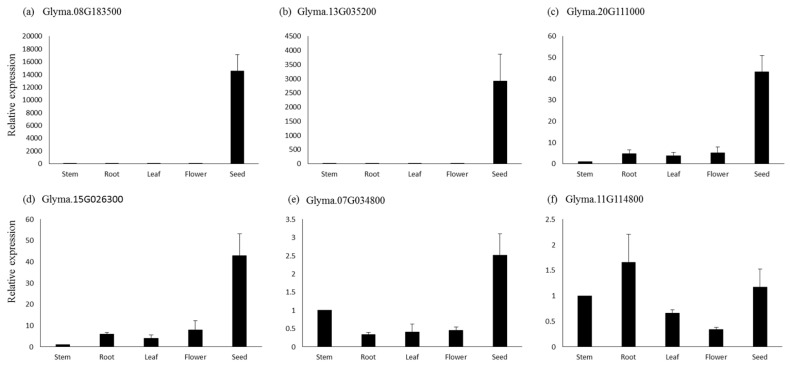
Quantitative real-time PCR analysis of six genes in different tissues of soybean. The relative expressions for all genes were calculated using 2 −∆∆CT. Bars represent the standard errors of the means of three biological and technical replicates.

For qPCR analysis, six genes were selected based on their higher expressions in seeds and other seed tissues. The genes *Glyma.08G183500,* which is a sugar efflux transporter sweet 24, and *Glyma.13G035200,* involved in tyrosine metabolism, showed seed-specific expression, while their expression was negligible in the other tissues based on the qPCR analysis (Figure 7). Similarly, genes *Glyma.15G026300* (*lipoxygenase1*) and *Glyma.20G111000* (*fatty acid desaturase*) showed several-fold higher expressions in seed tissues compared to the flower, root, and leaf, respectively. The gene *Glyma.07G034800* (*lipoxygenase*) also showed higher expression in seed tissue, followed by flower, leaf, and root. The genes *Glyma.11G114800* (*bZIP* transcription factor) showed the highest expression in roots, followed by seed, leaf, and flower (Figure 7). Similar expression patterns of these genes were also observed in the RNA-seq data.

## 4. Discussion

The soybean seed oil and protein content are complex traits and have a negative and positive correlation with other agronomically important traits. Many studies exploring QTL analysis using different bi-parental mapping populations have been performed in soybeans. However, only a few specific regions or genes governing the seed oil content have been identified. The soybean oil and protein content are well-studied traits in different environments and genetic backgrounds. Therefore, meta-QTLs can benefit the identification of QTLs associated with multiple environments and genetic backgrounds. Meta-QTL analysis was performed for the identification of consensus loci governing seed oil and protein content, and to narrow down the range of confidence intervals. In this study, the consensus positions of linked markers were used instead of the QTL positions mentioned in the previous studies. The marker position in a mapping study is based on recombination events between adjacent markers, and links a marker to a trait or QTL position, depending upon other factors such as population size and types, numbers of molecular markers, and many other factors. Therefore, markers and QTL positions vary among different studies. The consensus map used in this study provides positions of QTLs with high accuracy. Previously, some studies have been carried out for the meta-analysis of soybeans for different traits, including oil and protein content, and have identified many significant meta-QTLs. Similarly, a meta-analysis implementing the statistical methods provided by Goffinet and Gerber [28] was performed to increase the accuracy and precision of meta-QTLs. We have projected 428 out of 509 QTLs for oil content and 436 out of 460 QTLs for protein content onto the reference map, and integrated them into 55 (oil) and 58 (protein) meta-QTLs, including many minor QTLs of oil and protein content. Only 14 significant meta-QTLs were selected for oil and 16 for protein content possessing more than ten projected QTLs. Many studies have reported meta-QTLs in close proximity to those identified in the present study [19,28,29,30]. Previously, Qi et al (2018) [30] used about 313 QTLs for oil and 231 QTLs for protein. The maximum of 21 and 13 QTLs were projected by Qi et al (2018) [30] on chromosome 20 for oil and protein content, respectively. In the present study, 26 and 37 QTLs for seed oil and protein content were projected on chromosome 20.

A total of 458 candidate genes related to fatty acid/protein bio-synthesis/degradation, transporters and transcriptional factors were identified within all the meta-QTLs identified in the present study. The MetaQTL-20-2 with the most projected QTLs has 36 genes related to lipid and protein metabolism. The other identified genes are involved in various processes, such as TAG degradation, sucrose biosynthesis, acyl carrier, Cytochrome P450, and transcription factors. The roles of various transcriptional factors in regulating lipid regulation have been studied. The maximum number of 32 genes related to transcriptional factors, including Far1, Myb, WRKY, and AP2, were present on MetaQTL-PC_18.3, followed by 25 transcription factors genes in MetaQTL-PC_13.3. The Myb transcription factor was reported to associate with oil accumulation in Arabidopsis [31]. The AP2/ERF and R2R3/MYB transcription factors regulate the lipid metabolic process in response to temperature stress [32]. The Wrinkled gene encoding the AP2/EREB gene regulates the late glycolysis process and the plastid fatty acid biosynthesis pathways [33]. The high expression of the WRKY6 gene in developing seeds of Arabidopsis has a specified role in the dynamics of seed oil content [34]. The FAR1 (far-red-impaired response) transcription factors were reported to be associated with the circadian clock [35]. FAR1 is also known to control shoot development, chlorophyll biosynthesis, starch, and ABA synthesis [36,37,38]. The seeds act as a reservoir of nutrients and cannot synthesize all the precursor biomolecules. Therefore, the transporter proteins in seeds play an important role in increasing the seed nutrient contents. These transporter proteins provide the raw materials to the seed. The ABC, lipid, amino acid and sugar transporters were identified in the meta-QTL regions. The ABC transporter is known to transport fatty acid/lipids [39,40]. The sugar is mainly synthesized in leaves by the process of photosynthesis [41] and then transported to different storage tissues in the form of sucrose [41]. In seeds, sucrose is the main competitive and key carbon source for the biosynthesis of different biomolecules, and might be responsible for the negative correlation between the traits. Around 12 genes related to sucrose biosynthetic/degradation pathways were identified in hotspot meta-QTLs. Different sucrose transporters translocate the sugar to different sink tissues; hence, these sugar transporters play an important role in the improvement of seed traits. A total of 54 sugar transporters were identified on all meta-QTLs. The allelic variations in sugar transporter were reported to be associated with seed oil content in soybean [42]. The mutated sugar transporter in soybean embryo resulted in retarded growth, indicating the importance of sugar transporters [43]. These transporter proteins are very pivotal to plant development. In Arabidopsis, the mutation in three sugar transporter causes a drastic effect on seed sizes, shapes, and lipid contents. Sugar transporter overexpression and knockout resulted in large and small plant sizes in Arabidopsis [44]. Two sugar transporter genes (*Glyma.08G183500* and *Glyma.15G049200*) were highly expressed in developing seeds and parenchyma cells of the seed coat in the present study. The allelic variation and selection of sugar transporter genes during the domestication resulted in oil and protein improvement [42,45]. Some of the lipid transporter genes showed high differential expression in developing seeds in the present study. The mutated lipid transporter gene was reported to decrease the fatty acid content in rice seeds [46].

Several genes involved in different steps of the fatty acid pathways, such as 3-ketoacyl-CoA synthase, acyl-[acyl-carrier-protein] desaturase, Acyl carrier protein, fatty acid desaturase, very-long-chain 3-oxoacyl-CoA synthase, and long-chain acyl-CoA synthetase, were identified on different meta-QTLs. The very-long-chain 3-oxoacyl-CoA synthase proteins are acyl-activating enzymes involved in fatty acid transport from plastid to ER for further elongation and triacylglycerol (TAG) formation. The seven malate transporter proteins identified on different meta-QTLs play an important role in the uptake of phosphate and nitrate elements. The malate and other organic acids were used as precursors for amino-acid biosynthesis and as temporary carbon storage in CAM plants. The malate transporters (OsALMT7) were reported to maintain panicle size and grain yield in rice [47]. Thus, the malate transporter plays an important role in nutrient accumulation. The pyruvate dehydrogenase kinase has an important role in the regulation of acetyl CoA, a precursor molecule for fatty acid synthesis, and NADH. The knockdown of pyruvate dehydrogenase kinase resulted in a higher accumulation of TAG in Arabidopsis [48] and alga *Nannochloropsis salina* [49]. Many other dehydrogenase enzymes involved in the tricarboxylic acid (TCA) cycle were also identified, including succinate dehydrogenase, malate dehydrogenase, aldehyde dehydrogenase, and other alcohol dehydrogenases. Several enzymes specific to chloroplast and involved in the assimilation of carbon during photosynthesis were identified in the meta-QTL regions. Several genes were chloroplast-specific, and might play an important role in the biosynthetic process. Many genes related to amino acid/protein synthesis were identified on meta-QTL regions, including the asparagine synthetase, cysteinyl-tRNA synthetase, and late embryogenesis abundant protein encoding gene, which accumulates during maturity in seed development. Several genes related to oil and protein degradation have been identified on meta-QTLs. The genes lipoxygenase, TAG lipase, GDSL esterase, lysosomal acid lipase, fatty acyl-CoA reductase, glutamate dehydrogenase, proline dehydrogenase, and methylmalonate-semialdehyde dehydrogenase were identified in the present study. Lipoxygenase plays a very important role in plant physiology and defense. The lipoxygenase involved in jasmonic acid biosynthesis utilizes fatty acids.

Recently, superior haplotypes were utilized in breeding programs for crop improvement. In haplotype-based breeding, the breeder utilizes the genetic variation of candidate genes. Many studies have reported the superior haplotypes for salt tolerance [50] and plant height [51] in soybean, and grain quality in rice [52]. In our study, significant haplotypic associations were observed for fatty acid desaturase (FAD2-1B) genes with six haplotypes (Hap1 to Hap6). Haplotype Hap5 with two non-synonymous mutations manifests a significant change in oil and protein content. Similarly, the haplotype analysis of the sugar transporter (*Glyma.15g049200*) gene present in MetaQTL-PC15.2 showed a significant association with oil and protein content in soybean [42,45]. Further analysis and validation of other candidate genes are required to find the superior haplotypes responsible for the seed oil and protein contents in more diverse germplasms.

Based on the functional annotations, expression and missense mutations, 94 genes were selected as the most probable candidates regulating seed oil and protein content. Previously, [29] selected only 67 functional candidate genes within the nine identified meta-QTL regions, excluding transporters and transcription factors. The expression analysis revealed that the probable genes within the identified region were expressed in developing seeds, mostly in the parenchyma cell of the seed coat, epidermis, and different tissues of the developing embryo. The higher expression of candidate genes, specifically in seed tissues, indicates their role in oil and protein biosynthetic pathways. Recently, *Glyma.15g049200* was identified as a candidate gene in a mapping population of chromosome segment substitution lines for a quantitative trait locus (QTL) controlling seed oil content [53]. In this study, we have found two sugar transporters (*Glyma.08G183500* and *Glyma.15G049200*) predominantly expressed in seed tissues. The sugar transporter is involved in the transportation of sugar to sink tissue, where it is utilized for biomolecule synthesis [43,44]. The knockout and overexpression of the sugar transporter have resulted in contrasting phenotypes for seed size, oil and protein content [54], which suggests an important role in providing raw material to developing seeds for oil and protein accumulation. The lipoxygenases (*Glyma.07G034800* and *Glyma.15G026300*) were found to be highly specific to late development/dry soybean seeds, and these genes are responsible for the oxidation of polyunsaturated fatty acid. The high expression of lipoxygenase in dry seeds suggests their role in lipid degradation. The fatty acid desaturase *FAD2-1B* (*Glyma.20G111000*) showed expression from the early to the mid-mature development stages of soybean seeds. Mutations in this gene are known to enhance oleic acid content. The candidate genes with higher expressions in seeds and possessing nonsense mutations can be prioritized for further functional validation studies through approaches such as multi-target genome editing. The functional validation of the selected genes will help us to understand their roles in the regulation of seed oil and protein contents in soybean.

## 5. Conclusions

The present study explored about a thousand previously reported QTLs for seed oil and protein content in soybean to determine meta-QTLs. The amount of QTL data used here is robust enough to identify meta-QTLs with high confidence and narrow intervals. The extensive evaluation of genes located at meta-QTLs led to the identification of promising candidate genes related to fatty acid and protein biosynthetic pathways. Of the significant meta-QTLs identified here, two meta-QTLs, one on chromosome 6 and the other on chromosome 20, comprised 26 QTLs each for oil and 37 QTLs for protein. Such a large number of QTLs included in these meta-QTLs made them the most promising candidates for subsequent region-specific targeted association analysis. The availability of whole genome resequencing information for 1511 diverse soybean genotypes provided an excellent opportunity to perform the targeted association analysis and haplotyping of candidate genes. The SNPs localized at meta-QTL on chromosomes 6 and 20 identified through region-specific association analysis will be helpful for marker-assisted breeding applications. Furthermore, the haplotype analysis revealed six haplotypes for the *FAD2-1B* (*Glyma.20g111000*) gene, which clearly distinguished the high oil haplotype (Hap1) group from that of the low oil haplotype (Hap5) group. The information of the superior haplotype of *FAD2-1B* and other genes for seed oil content can be further exploited to improve seed oil content in soybeans. Similarly, the present study also explored high-throughput transcriptome data for candidate gene prioritization. Seed-specific expression was observed for the gene *Glyma.08G183500* (sweet 24), which is a sugar efflux transporter, and *Glyma.13G035200,* involved in tyrosine metabolism and *FAD2-1B*, also showed high expression in the soybean seeds. The information of candidate genes provided here will serve as a starting point for gene characterization studies. More importantly, the narrowed, pinpointed meta-QTL regions will be helpful for map-based gene cloning and marker-assisted breeding.

## Figures and Tables

**Table 1:** Details of significant meta-quantitative trait loci (meta-QTLs) identified for seed oil and protein content in soybean.

MetaQTL ^1^	MetaQTL Position (cm)	CI^2^ (cm)	No of QTLs	Left Marker			Right Marker		
				Name	Map Position (cm)	Physical Position (bp)	Name	Map Position (cm)	Physical Position (bp)
METAQTL-OC_1.2	41.83	41.56–42.1	10	BARC-057643-14873	41.58	38765880	BARC-055131-13049	42.23	40218915
METAQTL-OC_2.4	117.27	116.81–117.74	13	BARC-054149-12354	118.34	45295687	Satt274	118.62	45267222
METAQTL-PC_3.1	28.08	27.72–28.45	13	BARC-016199-02307	25.97	5664735	BARC-044085-08610	27.18	7805399
METAQTL-OC_5.4	81.41	80.95–81.87	11	BARC-020535-04656	80.93	40294725	BARC-021775-04203	81.93	39224723
METAQTL-OC_6.2	30.46	30.32–30.6	10	Satt640	29.63	4682853	BARC-018563-02977	30.83	4789924
METAQTL-PC_6.1	38.74	38.05–39.43	15	Satt281	38.9	6529270	BARC-027948-06704	40.25	6712097
METAQTL-PC_6.3	109.02	108.46–109.58	16	BARC-028441-05872	108.33	47212988	Satt307	109.96	46820834
METAQTL-OC_6.4	108.54	108.52–108.57	26	BARC-010457-00640	108.5	45851263	BARC-062515-17881	108.55	46596066
METAQTL-PC_7.2	48.46	47.94–48.98	11	BARC-048517-10647	47.38	8461619	Satt245	49.03	9357922
METAQTL-PC_8.2	53.56	52.72–54.41	16	BARC-053809-12037	52.44	10179802	BARC-013587-01169	54.55	10563212
METAQTL-PC_9.2	43.74	43.34–44.15	15	BARC-041483-08020	43.33	32421233	BARC-050815-09887	44.17	33502306
METAQTL-OC_9.4	72.05	71.54–72.56	12	BARC-065467-19490	71.27	44006810	BARC-008211-00113	73.2	44329945
METAQTL-PC_10.2	54.94	54.31–55.57	14	BARC-059863-16170	54.35	9249171	BARC-055953-13923	54.63	9887514
METAQTL-PC_10.3	95.12	94.29–95.95	11	BARC-050013-09288	94.97	44718071	BARC-029627-06257	95.93	44695771
METAQTL-OC_12.4	83.71	82.87–84.56	10	BARC-040047-07645	82.95	35640928	BARC-017985-02493	84.21	36147908
METAQTL-PC_13.2	61.43	60.97–61.9	10	Satt335	61.05	32721481	BARC-055499-13329	61.35	32684846
METAQTL-PC_13.3	71.73	71.15–72.32	10	BARC-027502-06598	71.11	35434883	BARC-055229-13122	71.89	35948473
METAQTL-OC_13.3	73.51	72.87–74.15	15	BARC-055229-13122	71.89	35948473	BARC-052431-11446	74.2	36674201
METAQTL-OC_14.4	62.97	62.66–63.29	14	Satt020	62.76	41294144	Satt556	63.25	38859467
METAQTL-PC_15.2	18.2	17.75–18.66	14	BARC-042271-08229	19.5	3745486	BARC-042349-08247	19.8	3985288
METAQTL-PC_17.3	76.66	75.95–77.38	13	BARC-060095-16373	75.85	27299017	BARC-019497-03640	77.39	15462178
METAQTL-PC_18.3	58.41	57.37–59.45	10	Satt138	57.08	41530961	BARC-029457-06193	59.71	48057611
METAQTL-OC_19.2	47.98	47.51–48.46	13	BARC-016181-02303	46.51	38087635	BARC-060795-16881	48.45	39961359
METAQTL-PC_20.1	19.16	18.42–19.9	11	BARC-021887-04232	18.51	1900702	BARC-052017-11314	19.96	2103067
METAQTL-OC_20.1	19.23	18.9–19.56	13	BARC-027552-06609	18.91	1999670	BARC-042619-08314	19.68	2072947
METAQTL-OC_20.2	29.82	29.63–30.01	26	BARC-054889-12193	29.6	23009963	BARC-052445-11461	30.13	28391296
METAQTL-PC_20.2	29.94	29.82–30.06	37	Satt239	29.61	25275083	BARC-023131-03782	30	28349696
METAQTL-OC_20.3	44.34	44.18–44.51	17	BARC-039067-07437	44	35644777	BARC-055423-13277	44.95	36055353
METAQTL-PC_20.3	45.87	45.33–46.41	19	BARC-055423-13277	44.95	36055353	BARC-054275-12433	46.28	49323397
METAQTL-OC_20.4	75.43	74.34–76.53	18	BARC-041051-07902	74.61	40634800	BARC-029151-06100	76.68	41023496

^1^ In metaQTL name the OC code for oil content and PC code for protein content where the 2.4 code for chromosome 02 and fourth meta-QTL.

## Data Availability

Not applicable.

## Supplementary Materials

The following supporting information can be downloaded at: https://www.mdpi.com/article/10.3390/cells12010097/s1, Figure S1. The representation of meta-quantitative trait loci (meta-QTLs) of soybean seed oil content present on different chromosome. The rectangle with rounded ends represents chromosome, the different colors lines on left side represent the QTLs present in a meta-QTLs, right side of rectangle indicates the position (cM) and name of markers; Figure S2. The representation of meta-quantitative trait loci (meta-QTLs) of soybean seed protein content present on different chromosome. The rectangle with rounded ends represents chromosome, the different colors lines on left side represent the QTLs present in a meta-QTLs, right side of rectangle indicates the position (cM) and name of markers; Table S1. analysisList of quantitative trait loci (QTLs) reported for seed oil and protein content in different studies used for meta-QTL analysis in soybean; Table S2. List of genes and their primer sequences used for quantitative real-time PCR; Table S3. Details of meta-quantitative trait loci (meta-QTLs) identified for seed oil and protein content in soybean; Table S4. Significant SNPs associated with oil and protein content identified by different GWAS method; Table S5. Details of genes present within the meta-quantitative trait loci (meta-QTLs) regions of seed oil and protein content in soybean; Table S6. List of selected genes within the meta-QTLs related to lipid and protein metabolic pathway; Table S7. List of genes having missense and non-sense mutation along with their provean score; Table S8: The student t-test analysis of different haplotypes. The phenotype data of different haplotypes were compared to haplotypes with reference type allele; Table S9. Expression of selected candidate genes based on the RNAseq data related to seed oil and protein content in soybean.
