# Supplementary material for: Pinpointing Genomic Regions and Candidate Genes Associated with Seed Oil and Protein Content in Soybean through an Integrative Transcriptomic and QTL Meta-Analysis
Source: Cells. 2022 Dec 26;12(1):97. doi: 10.3390/cells12010097 (PMC9818467; doi:10.3390/cells12010097)
Supplement: Supplementary file 1 [file cells-12-00097-s001.zip › Supplementary Figures.pdf]

Figure S1. The representation of meta-quantitative trait loci (meta-QTLs) of soybean seed oil content present on different chromosome. The rectangle with rounded ends represent chromosome, the different colors lines on left side represent the QTLs present in a meta-QTLs, right side of rectangle indicates the position (cM) and name of markers.

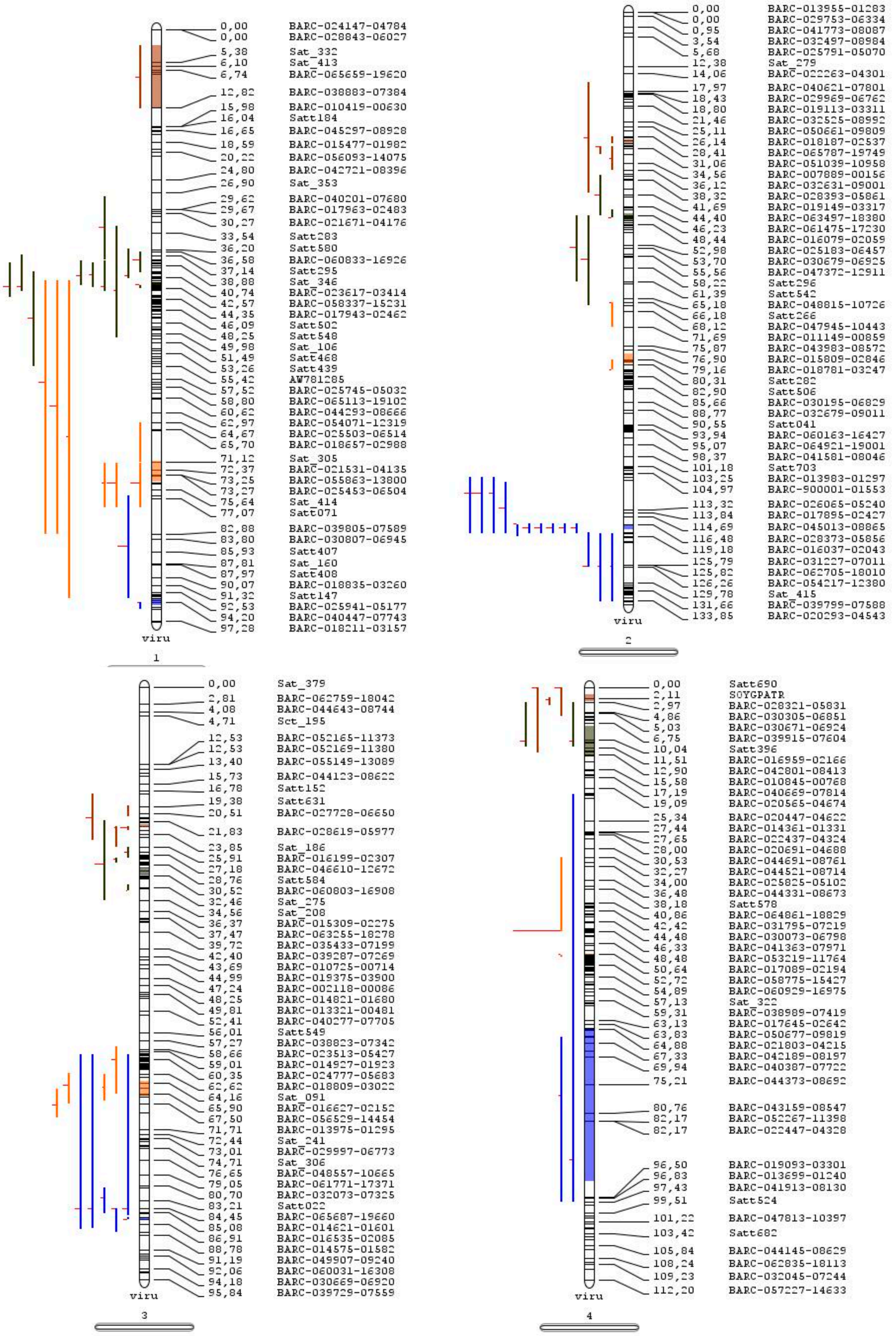

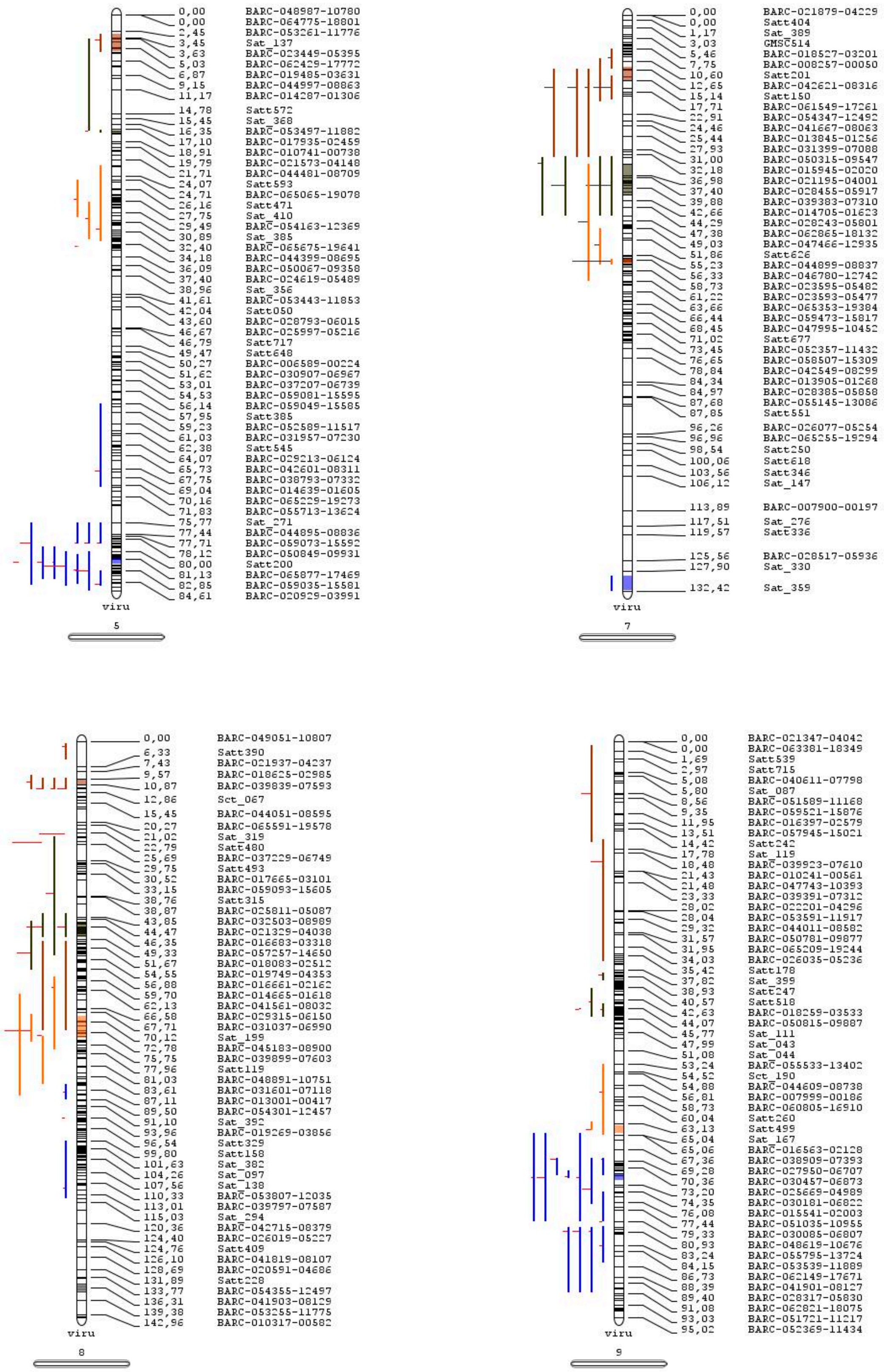
0,00 BARC-048987-10780
0,00 BARC-064775-18801
2,45 BARC-053261-11776
3,45 Sat_137
3,63 BARC-023449-05395
5,03 BARC-062429-17772
6,87 BARC-019485-03631
9,15 BARC-044997-08863
11,17 BARC-014287-01306
14,78 Satt572
15,45 Sat_368
16,35 BARC-053497-11882
17,10 BARC-017935-02459
18,91 BARC-010741-00738
19,79 BARC-021573-04148
21,71 BARC-044481-08709
24,07 Satt593
24,71 BARC-065065-19078
26,16 Satt471
27,75 Sat_410
29,49 BARC-054163-12369
30,89 Sat_385
32,40 BARC-065675-19641
34,18 BARC-044399-08695
36,09 BARC-050067-09358
37,40 BARC-024619-05489
38,96 Sat_356
41,61 BARC-053443-11853
42,04 Satt050
43,60 BARC-028793-06015
46,67 BARC-025997-05216
46,79 Satt717
49,47 Satt648
50,27 BARC-006589-00224
51,62 BARC-030907-06967
53,01 BARC-037207-06739
54,53 BARC-059081-15595
56,14 BARC-059049-15585
57,95 Satt385
59,23 BARC-052589-11517
61,03 BARC-031957-07230
62,38 Satt545
64,07 BARC-029213-06124
65,73 BARC-042601-08311
67,75 BARC-038793-07332
69,04 BARC-014639-01605
70,16 BARC-065229-19273
71,83 BARC-055713-13624
75,77 Sat_271
77,44 BARC-044895-08836
77,71 BARC-059073-15592
78,12 BARC-050849-09931
80,00 Satt200
81,13 BARC-065877-17469
82,85 BARC-059035-15581
84,61 BARC-020929-03991
viru
5
0,00 BARC-021879-04229
0,00 Satt404
1,17 Sat_389
3,03 GMSC514
5,46 BARC-018527-03201
7,75 BARC-008257-00050
10,60 Satt201
12,65 BARC-042621-08316
15,14 Satt150
17,71 BARC-061549-17261
22,91 BARC-054347-12492
24,46 BARC-041667-08063
25,44 BARC-013845-01256
27,93 BARC-031399-07088
31,00 BARC-050315-09547
32,18 BARC-015945-02020
36,98 BARC-021195-04001
37,40 BARC-028455-05917
39,88 BARC-039383-07310
42,66 BARC-014705-01623
44,29 BARC-028243-05801
47,38 BARC-062865-18132
49,03 BARC-047466-12935
51,86 Satt626
55,23 BARC-044899-08837
56,33 BARC-046780-12742
58,73 BARC-023595-05482
61,22 BARC-023593-05477
63,66 BARC-065353-19384
66,44 BARC-059473-15817
68,45 BARC-047995-10452
71,02 Satt677
73,45 BARC-052357-11432
76,65 BARC-058507-15309
78,84 BARC-042549-08299
84,34 BARC-013905-01268
84,97 BARC-028385-05858
87,68 BARC-055145-13086
87,85 Satt551
96,26 BARC-026077-05254
96,96 BARC-065255-19294
98,54 Satt250
100,06 Satt618
103,56 Satt346
106,12 Sat_147
113,89 BARC-007900-00197
117,51 Sat_276
119,57 Satt336
125,56 BARC-028517-05936
127,90 Sat_330
132,42 Sat_359
viru
7
0,00 BARC-049051-10807
6,33 Satt390
7,43 BARC-021937-04237
9,57 BARC-018625-02985
10,87 BARC-039839-07593
12,86 Sct_067
15,45 BARC-044051-08595
20,27 BARC-065591-19578
21,02 Sat_319
22,79 Satt480
25,69 BARC-037229-06749
29,75 Satt493
30,52 BARC-017665-03101
33,15 BARC-059093-15605
38,76 Satt315
38,87 BARC-025811-05087
43,85 BARC-032503-08989
44,47 BARC-021329-04038
46,35 BARC-016683-03318
49,33 BARC-057257-14650
51,67 BARC-018083-02512
54,55 BARC-019749-04353
56,88 BARC-016661-02162
59,70 BARC-014665-01618
62,13 BARC-041561-08032
66,58 BARC-029315-06150
67,71 BARC-031037-06990
70,12 Sat_199
72,78 BARC-045183-08900
75,75 BARC-039899-07603
77,96 Satt119
81,03 BARC-048891-10751
83,61 BARC-031601-07118
87,11 BARC-013001-00417
89,50 BARC-054301-12457
91,10 Sat_392
93,96 BARC-019269-03856
96,54 Satt329
99,80 Satt158
101,63 Sat_382
104,26 Sat_097
107,56 Sat_138
110,33 BARC-053807-12035
113,01 BARC-039797-07587
115,03 Sat_294
120,36 BARC-042715-08379
124,40 BARC-026019-05227
124,76 Satt409
126,10 BARC-041819-08107
128,69 BARC-020591-04686
131,89 Satt228
133,77 BARC-054355-12497
136,31 BARC-041903-08129
139,38 BARC-053255-11775
142,96 BARC-010317-00582
viru
8
0,00 BARC-021347-04042
0,00 BARC-063381-18349
1,69 Satt539
2,97 Satt715
5,08 BARC-040611-07798
5,80 Sat_087
8,56 BARC-051589-11168
9,35 BARC-059521-15876
11,95 BARC-016397-02579
13,51 BARC-057945-15021
14,42 Satt242
17,78 Sat_119
18,48 BARC-039923-07610
21,43 BARC-010241-00561
21,48 BARC-047743-10393
23,33 BARC-039391-07312
28,02 BARC-022201-04296
28,04 BARC-053591-11917
29,32 BARC-044011-08582
31,57 BARC-050781-09877
31,95 BARC-065209-19244
34,03 BARC-026035-05236
35,42 Satt178
37,82 Sat_399
38,93 Satt247
40,57 Satt518
42,63 BARC-018259-03533
44,07 BARC-050815-09887
45,77 Sat_111
47,99 Sat_043
51,08 Sat_044
53,24 BARC-055533-13402
54,52 Sct_190
54,88 BARC-044609-08738
56,81 BARC-007999-00186
58,73 BARC-060805-16910
60,04 Satt260
63,13 Satt499
65,04 Sat_167
65,06 BARC-016563-02128
67,36 BARC-038909-07393
69,28 BARC-027950-06707
70,36 BARC-030457-06873
73,20 BARC-025669-04989
74,35 BARC-030181-06822
76,08 BARC-015541-02003
77,44 BARC-051035-10955
79,33 BARC-030085-06807
80,93 BARC-048619-10676
83,24 BARC-055795-13724
84,15 BARC-053539-11889
86,73 BARC-062149-17671
88,39 BARC-041901-08127
89,40 BARC-028317-05830
91,08 BARC-062821-18075
93,03 BARC-051721-11217
95,02 BARC-052369-11434
viru
9

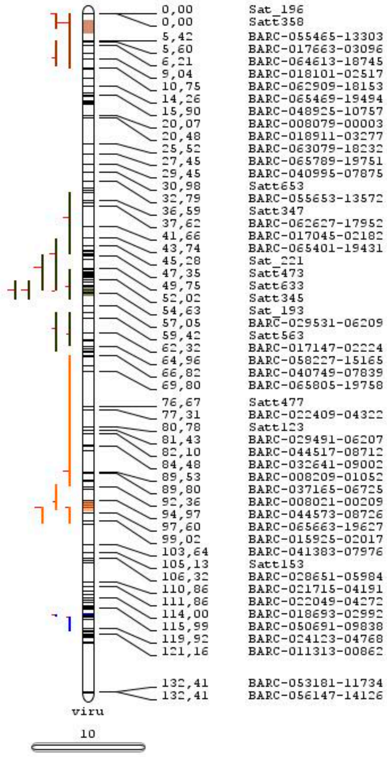
0,00 Sat_196
0,00 Satt358
5,42 BARC-055465-13303
5,60 BARC-017663-03096
6,21 BARC-064613-18745
9,04 BARC-018101-02517
10,75 BARC-062909-18153
14,26 BARC-065469-19494
15,90 BARC-048925-10757
20,07 BARC-008079-00003
20,48 BARC-018911-03277
25,52 BARC-063079-18232
27,45 BARC-065789-19751
29,45 BARC-040995-07875
30,98 Satt653
32,79 BARC-055653-13572
36,59 Satt347
37,62 BARC-062627-17952
41,66 BARC-017045-02182
43,74 BARC-065401-19431
45,28 Sat_221
47,35 Satt473
49,75 Satt633
52,02 Satt345
54,63 Sat_193
57,05 BARC-029531-06209
59,42 Satt563
62,32 BARC-017147-02224
64,96 BARC-058227-15165
66,82 BARC-040749-07839
69,80 BARC-065805-19758
76,67 Satt477
77,31 BARC-022409-04322
80,78 Satt123
81,43 BARC-029491-06207
82,10 BARC-044517-08712
84,48 BARC-032641-09002
89,53 BARC-008209-01052
89,80 BARC-037165-06725
92,36 BARC-008021-00209
94,97 BARC-044573-08726
97,60 BARC-065663-19627
99,02 BARC-015925-02017
103,64 BARC-041383-07976
105,13 Satt153
106,32 BARC-028651-05984
110,86 BARC-021715-04191
111,86 BARC-022049-04272
114,00 BARC-018693-02992
115,99 BARC-050691-09838
119,92 BARC-024123-04768
121,16 BARC-011313-00862
132,41 BARC-053181-11734
132,41 BARC-056147-14126
viru
10

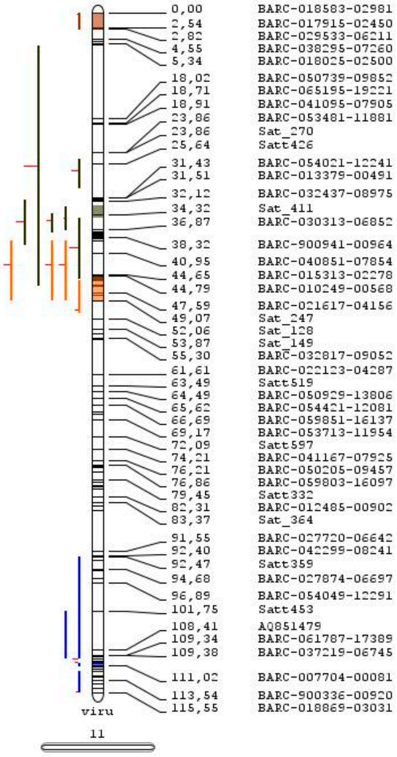
0,00 BARC-018583-02981
2,54 BARC-017915-02450
2,82 BARC-029533-06211
4,55 BARC-038295-07260
5,34 BARC-018025-02500
18,02 BARC-050739-09852
18,71 BARC-065195-19221
18,91 BARC-041095-07905
23,86 BARC-053481-11881
23,86 Sat_270
25,64 Satt426
31,43 BARC-054021-12241
31,51 BARC-013379-00491
32,12 BARC-032437-08975
34,32 Sat_411
36,87 BARC-030313-06852
38,32 BARC-900941-00964
40,95 BARC-040851-07854
44,65 BARC-015313-02278
44,79 BARC-010249-00568
47,59 BARC-021617-04156
49,07 Sat_247
52,06 Sat_128
53,87 Sat_149
55,30 BARC-032817-09052
61,61 BARC-022123-04287
63,49 Satt519
64,49 BARC-050929-13806
65,62 BARC-054421-12081
66,69 BARC-059851-16137
69,17 BARC-053713-11954
72,09 Satt597
74,21 BARC-041167-07925
76,21 BARC-050205-09457
76,86 BARC-059803-16097
79,45 Satt332
82,31 BARC-012485-00902
83,37 Sat_364
91,55 BARC-027720-06642
92,40 BARC-042299-08241
92,47 Satt359
94,68 BARC-027874-06697
96,89 BARC-054049-12291
101,75 Satt453
108,41 AQ851479
109,34 BARC-061787-17389
109,38 BARC-037219-06745
111,02 BARC-007704-00081
113,54 BARC-900336-00920
115,55 BARC-018869-03031
viru
11

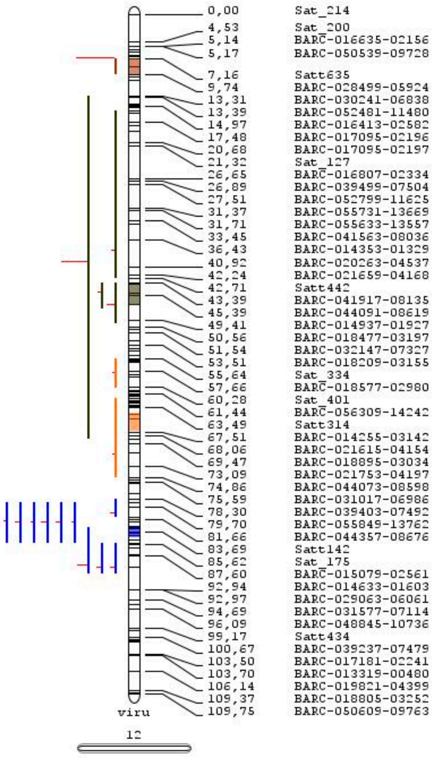
0,00 Sat_214
4,53 Sat_200
5,14 BARC-016635-02156
5,17 BARC-050539-09728
7,16 Satt635
9,74 BARC-028499-05924
13,31 BARC-030241-06838
13,39 BARC-052481-11480
14,97 BARC-016413-02582
17,48 BARC-017095-02196
20,68 BARC-017095-02197
21,32 Sat_127
26,65 BARC-016807-02334
26,89 BARC-039499-07504
27,51 BARC-052799-11625
31,37 BARC-055731-13669
31,71 BARC-055633-13557
33,45 BARC-041563-08036
36,43 BARC-014353-01329
40,92 BARC-020263-04537
42,24 BARC-021659-04168
42,71 Satt442
43,39 BARC-041917-08135
45,39 BARC-044091-08619
49,41 BARC-014937-01927
50,56 BARC-018477-03197
51,54 BARC-032147-07327
53,51 BARC-018209-03155
55,64 Sat_334
57,66 BARC-018577-02980
60,28 Sat_401
61,44 BARC-056309-14242
63,49 Satt314
67,51 BARC-014255-03142
68,06 BARC-021615-04154
69,47 BARC-018895-03034
73,09 BARC-021753-04197
74,86 BARC-044073-08598
75,59 BARC-031017-06986
78,30 BARC-039403-07492
79,70 BARC-055849-13762
81,66 BARC-044357-08676
83,69 Satt142
85,62 Sat_175
87,60 BARC-015079-02561
92,94 BARC-014633-01603
92,97 BARC-029063-06061
94,69 BARC-031577-07114
96,09 BARC-048845-10736
99,17 Satt434
100,67 BARC-039237-07479
103,50 BARC-017181-02241
103,70 BARC-013319-00480
106,14 BARC-019821-04399
109,37 BARC-018805-03252
109,75 BARC-050609-09763
viru
12

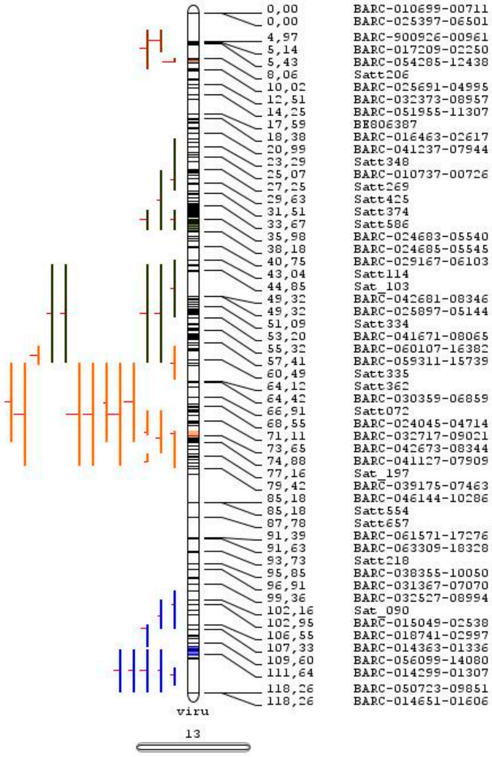
0,00 BARC-010699-00711
0,00 BARC-025397-06501
4,97 BARC-900926-00961
5,14 BARC-017209-02250
5,43 BARC-054285-12438
8,06 Satt206
10,02 BARC-025691-04995
12,51 BARC-032373-08957
14,25 BARC-051955-11307
17,59 BE806387
18,38 BARC-016463-02617
20,99 BARC-041237-07944
23,29 Satt348
25,07 BARC-010737-00726
27,25 Satt269
29,63 Satt425
31,51 Satt374
33,67 Satt586
35,98 BARC-024683-05540
38,18 BARC-024685-05545
40,75 BARC-029167-06103
43,04 Satt114
44,85 Sat_103
49,32 BARC-042681-08346
49,32 BARC-025897-05144
51,09 Satt334
53,20 BARC-041671-08065
55,32 BARC-060107-16382
57,41 BARC-059311-15739
60,49 Satt335
64,12 Satt362
64,42 BARC-030359-06859
66,91 Satt072
68,55 BARC-024045-04714
71,11 BARC-032717-09021
73,65 BARC-042673-08344
74,88 BARC-041127-07909
77,16 Sat_197
79,42 BARC-039175-07463
85,18 BARC-046144-10286
85,18 Satt554
87,78 Satt657
91,39 BARC-061571-17276
91,63 BARC-063309-18328
93,73 Satt218
95,85 BARC-038355-10050
96,91 BARC-031367-07070
99,36 BARC-032527-08994
102,16 Sat_090
102,95 BARC-015049-02538
106,55 BARC-018741-02997
107,33 BARC-014363-01336
109,60 BARC-056099-14080
111,64 BARC-014299-01307
118,26 BARC-050723-09851
118,26 BARC-014651-01606
viru
13

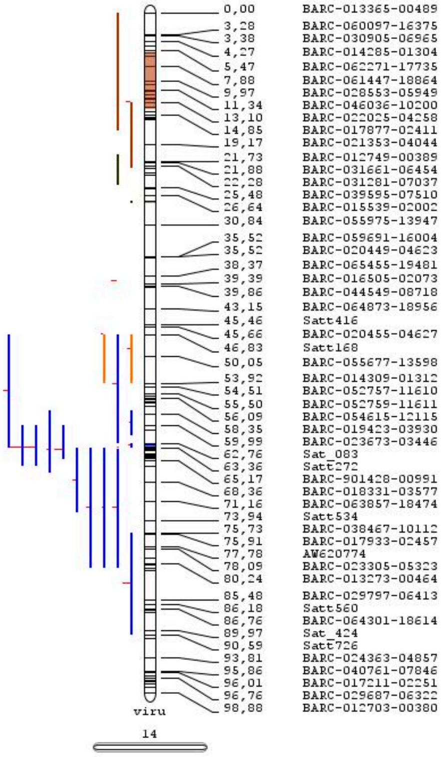
0,00 BARC-013365-00489
3,28 BARC-060097-16375
3,38 BARC-030905-06965
4,27 BARC-014285-01304
5,47 BARC-062271-17735
7,88 BARC-061447-18864
9,97 BARC-028553-05949
11,34 BARC-046036-10200
13,10 BARC-022025-04258
14,85 BARC-017877-02411
19,17 BARC-021353-04044
21,73 BARC-012749-00389
21,88 BARC-031661-06454
22,28 BARC-031281-07037
25,48 BARC-039595-07510
26,64 BARC-015539-02002
30,84 BARC-055975-13947
35,52 BARC-059691-16004
35,52 BARC-020449-04623
38,37 BARC-065455-19481
39,39 BARC-016505-02073
39,86 BARC-044549-08718
43,15 BARC-064873-18956
45,46 Satt416
45,66 BARC-020455-04627
46,83 Satt168
50,05 BARC-055677-13598
53,92 BARC-014309-01312
54,51 BARC-052757-11610
55,50 BARC-052759-11611
56,09 BARC-054615-12115
58,35 BARC-019423-03930
59,99 BARC-023673-03446
62,76 Sat_083
63,36 Satt272
65,17 BARC-901428-00991
68,36 BARC-018331-03577
71,16 BARC-063857-18474
73,94 Satt534
75,73 BARC-038467-10112
75,91 BARC-017933-02457
77,78 AW620774
78,09 BARC-023305-05323
80,24 BARC-013273-00464
85,48 BARC-029797-06413
86,18 Satt560
86,76 BARC-064301-18614
89,97 Sat_424
90,59 Satt726
93,81 BARC-024363-04857
95,86 BARC-040761-07846
96,01 BARC-017211-02251
96,76 BARC-029687-06322
98,88 BARC-012703-00380
viru
14

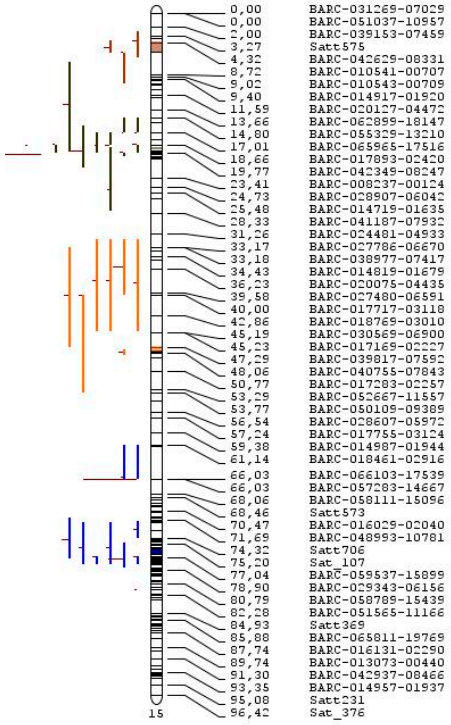
0,00 BARC-031269-07029
0,00 BARC-051037-10957
2,00 BARC-039153-07459
3,27 Satt575
4,32 BARC-042629-08331
8,72 BARC-010541-00707
9,02 BARC-010543-00709
9,40 BARC-014917-01920
11,59 BARC-020127-04472
13,66 BARC-062899-18147
14,80 BARC-055329-13210
17,01 BARC-065965-17516
18,66 BARC-017893-02420
19,77 BARC-042349-08247
23,41 BARC-008237-00124
24,73 BARC-028907-06042
25,48 BARC-014719-01635
28,33 BARC-041187-07932
31,26 BARC-024481-04933
33,17 BARC-027786-06670
33,18 BARC-038977-07417
34,43 BARC-014819-01679
36,23 BARC-020075-04435
39,58 BARC-027480-06591
40,00 BARC-017717-03118
42,86 BARC-018769-03010
45,19 BARC-030569-06900
45,23 BARC-017169-02227
47,29 BARC-039817-07592
48,06 BARC-040755-07843
50,77 BARC-017283-02257
53,29 BARC-052667-11557
53,77 BARC-050109-09389
56,54 BARC-028607-05972
57,24 BARC-017755-03124
59,38 BARC-014987-01944
61,14 BARC-018461-02916
66,03 BARC-066103-17539
66,03 BARC-057283-14667
68,06 BARC-058111-15096
68,46 Satt573
70,47 BARC-016029-02040
71,69 BARC-048993-10781
74,32 Satt706
75,20 Sat_107
77,04 BARC-059537-15899
78,90 BARC-029343-06156
80,79 BARC-058789-15439
82,28 BARC-051565-11166
84,93 Satt369
85,88 BARC-065811-19769
87,74 BARC-016131-02290
89,74 BARC-013073-00440
91,30 BARC-042937-08466
93,35 BARC-014957-01937
95,08 Satt231
96,42 Sat_376
15

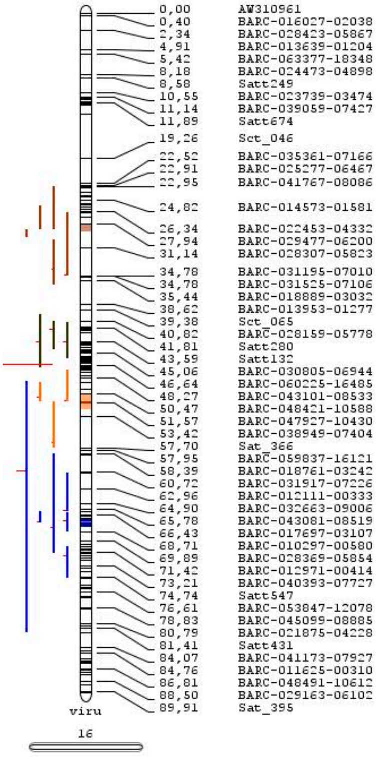
0,00 AW310961
0,40 BARC-016027-02038
2,34 BARC-028423-05867
4,91 BARC-013639-01204
5,42 BARC-063377-18348
8,18 BARC-024473-04898
8,58 Satt249
10,55 BARC-023739-03474
11,14 BARC-039059-07427
11,89 Satt674
19,26 Sct_046
22,52 BARC-035361-07166
22,91 BARC-025277-06467
22,95 BARC-041767-08086
24,82 BARC-014573-01581
26,34 BARC-022453-04332
27,94 BARC-029477-06200
31,14 BARC-028307-05823
34,78 BARC-031195-07010
34,78 BARC-031525-07106
35,44 BARC-018889-03032
38,62 BARC-013953-01277
39,38 Sct_065
40,82 BARC-028159-05778
41,81 Satt280
43,59 Satt132
45,06 BARC-030805-06944
46,64 BARC-060225-16485
48,27 BARC-043101-08533
50,47 BARC-048421-10588
51,57 BARC-047927-10430
53,42 BARC-038949-07404
57,70 Sat_366
57,95 BARC-059837-16121
58,39 BARC-018761-03242
60,72 BARC-031917-07226
62,96 BARC-012111-00333
64,90 BARC-032663-09006
65,78 BARC-043081-08519
66,43 BARC-017697-03107
68,71 BARC-010297-00580
69,89 BARC-028369-05854
71,42 BARC-012971-00414
73,21 BARC-040393-07727
74,74 Satt547
76,61 BARC-053847-12078
78,83 BARC-045099-08885
80,79 BARC-021875-04228
81,41 Satt431
84,07 BARC-041173-07927
84,76 BARC-011625-00310
86,81 BARC-048491-10612
88,50 BARC-029163-06102
89,91 Sat_395
viru
16

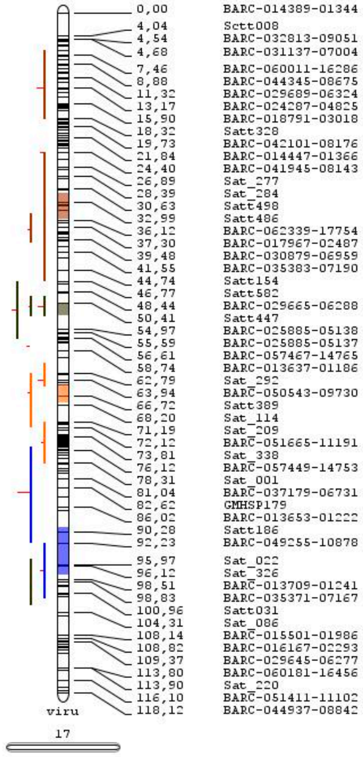
0,00 BARC-014389-01344
4,04 Sctt008
4,54 BARC-032813-09051
4,68 BARC-031137-07004
7,46 BARC-060011-16286
8,88 BARC-044345-08675
11,32 BARC-029689-06324
13,17 BARC-024287-04825
15,90 BARC-018791-03018
18,32 Satt328
19,73 BARC-042101-08176
21,84 BARC-014447-01366
24,40 BARC-041945-08143
26,89 Sat_277
28,39 Sat_284
30,63 Satt498
32,99 Satt486
36,12 BARC-062339-17754
37,30 BARC-017967-02487
39,48 BARC-030879-06959
41,55 BARC-035383-07190
44,74 Satt154
46,77 Satt582
48,44 BARC-029665-06288
50,41 Satt447
54,97 BARC-025885-05138
55,59 BARC-025885-05137
56,61 BARC-057467-14765
58,74 BARC-013637-01186
62,79 Sat_292
63,94 BARC-050543-09730
66,72 Satt389
68,20 Sat_114
71,19 Sat_209
72,12 BARC-051665-11191
73,81 Sat_338
76,12 BARC-057449-14753
78,31 Sat_001
81,04 BARC-037179-06731
82,62 GMHSP179
86,02 BARC-013653-01222
90,28 Satt186
92,23 BARC-049255-10878
95,97 Sat_022
96,12 Sat_326
98,51 BARC-013709-01241
98,83 BARC-035371-07167
100,96 Satt031
104,31 Sat_086
108,14 BARC-015501-01986
108,82 BARC-016167-02293
109,37 BARC-029645-06277
113,80 BARC-060181-16456
113,90 Sat_220
116,10 BARC-051411-11102
118,12 BARC-044937-08842
viru
17

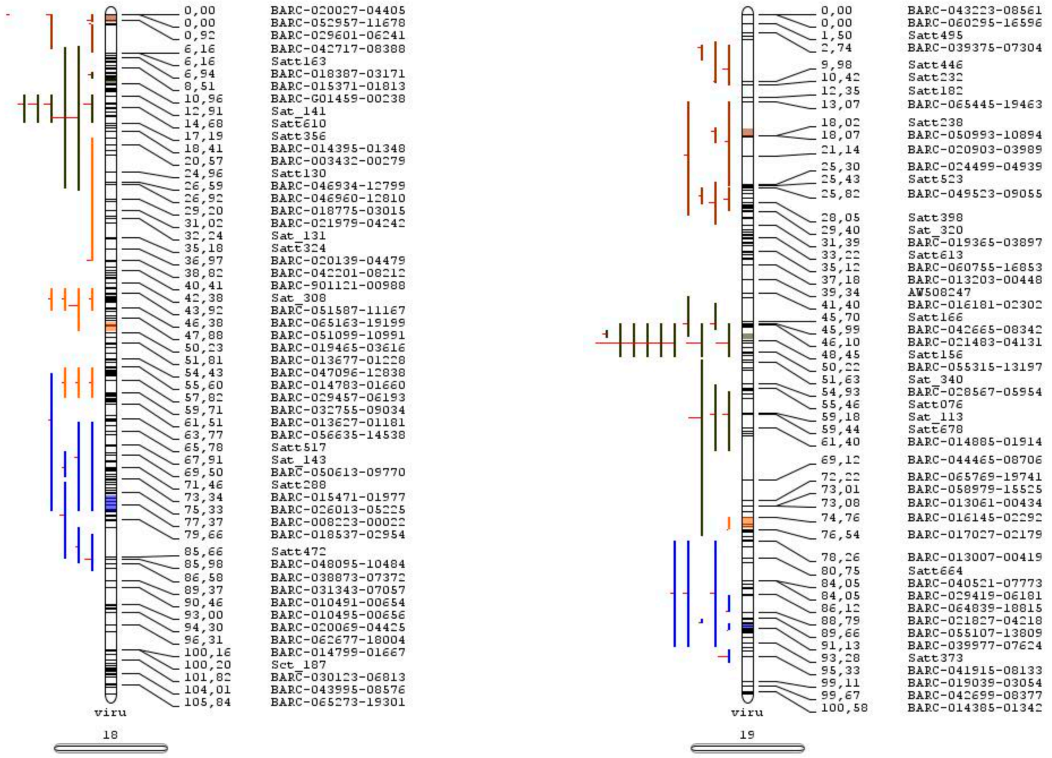
0,00 BARC-020027-04405
0,00 BARC-052957-11678
0,92 BARC-029601-06241
6,16 BARC-042717-08388
6,16 Satt163
6,94 BARC-018387-03171
8,51 BARC-015371-01813
10,96 BARC-G01459-00238
12,91 Sat_141
14,68 Satt610
17,19 Satt356
18,41 BARC-014395-01348
20,57 BARC-003432-00279
24,96 Satt130
26,59 BARC-046934-12799
26,92 BARC-046960-12810
29,20 BARC-018775-03015
31,02 BARC-021979-04242
32,24 Sat_131
35,18 Satt324
36,97 BARC-020139-04479
38,82 BARC-042201-08212
40,41 BARC-901121-00988
42,38 Sat_308
43,92 BARC-051587-11167
46,38 BARC-065163-19199
47,88 BARC-051099-10991
50,23 BARC-019465-03616
51,81 BARC-013677-01228
54,43 BARC-047096-12838
55,60 BARC-014783-01660
57,82 BARC-029457-06193
59,71 BARC-032755-09034
61,51 BARC-013627-01181
63,77 BARC-056635-14538
65,78 Satt517
67,91 Sat_143
69,50 BARC-050613-09770
71,46 Satt288
73,34 BARC-015471-01977
75,33 BARC-026013-05225
77,37 BARC-008223-00022
79,66 BARC-018537-02954
85,66 Satt472
85,98 BARC-048095-10484
86,58 BARC-038873-07372
89,37 BARC-031343-07057
90,46 BARC-010491-00654
93,00 BARC-010495-00656
94,30 BARC-020069-04425
96,31 BARC-062677-18004
100,16 BARC-014799-01667
100,20 Sct_187
101,82 BARC-030123-06813
104,01 BARC-043995-08576
105,84 BARC-065273-19301
viru
18
0,00 BARC-043223-08561
0,00 BARC-060295-16596
1,50 Satt495
2,74 BARC-039375-07304
9,98 Satt446
10,42 Satt232
12,35 Satt182
13,07 BARC-065445-19463
18,02 Satt238
18,07 BARC-050993-10894
21,14 BARC-020903-03989
25,30 BARC-024499-04939
25,43 Satt523
25,82 BARC-049523-09055
28,05 Satt398
29,40 Sat_320
31,39 BARC-019365-03897
33,22 Satt613
35,12 BARC-060755-16853
37,18 BARC-013203-00448
39,34 AW508247
41,40 BARC-016181-02302
45,70 Satt166
45,99 BARC-042665-08342
46,10 BARC-021483-04131
48,45 Satt156
50,22 BARC-055315-13197
51,63 Sat_340
54,93 BARC-028567-05954
55,46 Satt076
59,18 Sat_113
59,44 Satt678
61,40 BARC-014885-01914
69,12 BARC-044465-08706
72,22 BARC-065769-19741
73,01 BARC-058979-15525
73,08 BARC-013061-00434
74,76 BARC-016145-02292
76,54 BARC-017027-02179
78,26 BARC-013007-00419
80,75 Satt664
84,05 BARC-040521-07773
84,05 BARC-029419-06181
86,12 BARC-064839-18815
88,79 BARC-021827-04218
89,66 BARC-055107-13809
91,13 BARC-039977-07624
93,28 Satt373
95,33 BARC-041915-08133
99,11 BARC-019039-03054
99,67 BARC-042699-08377
100,58 BARC-014385-01342
viru
19

Figure S2. The representation of meta-quantitative trait loci (meta-QTLs) of soybean seed protein content present on different chromosome. The rectangle with rounded ends represent chromosome, the different colors lines on left side represent the QTLs present in a meta-QTLs, right side of rectangle indicates the position (cM) and name of markers.

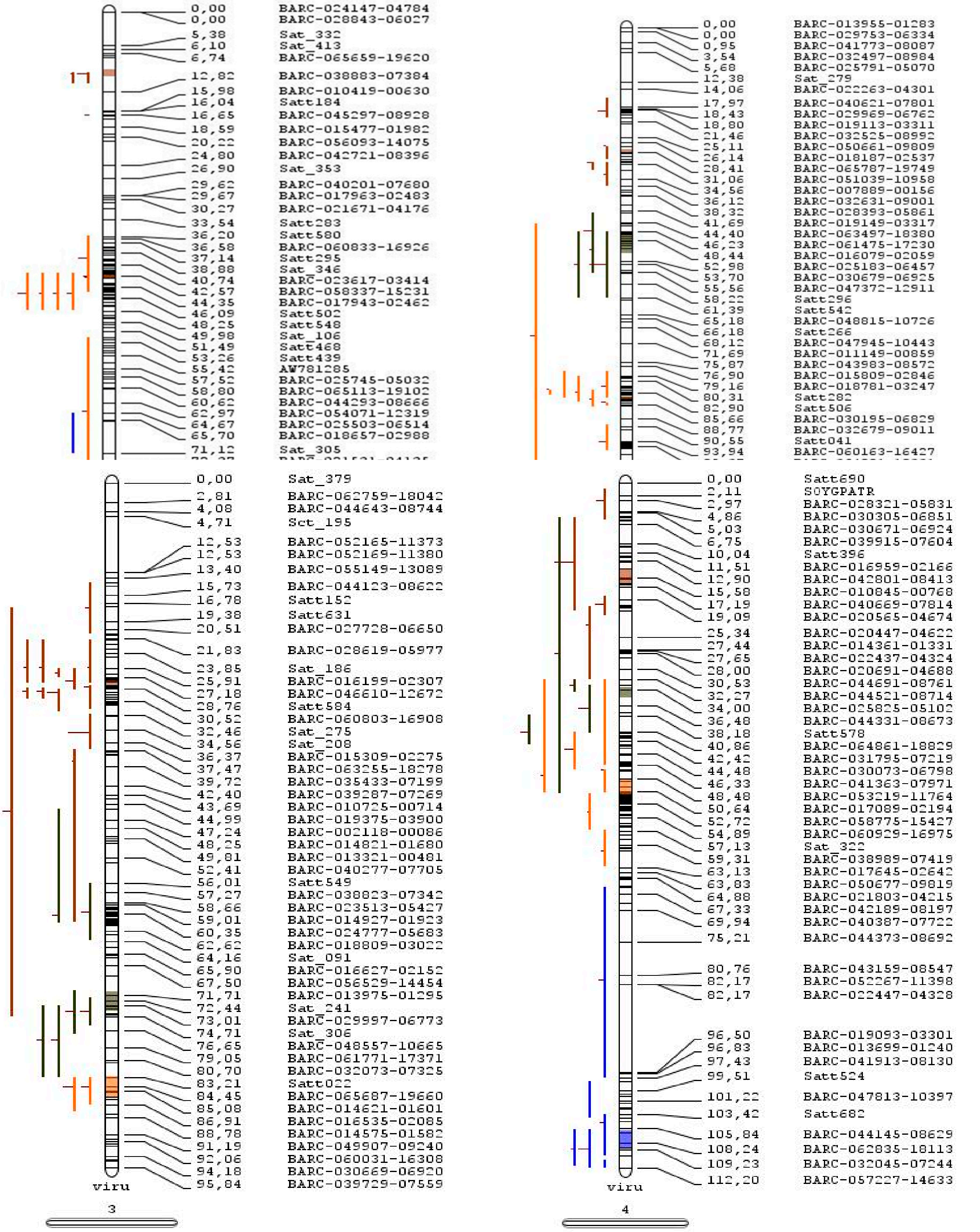

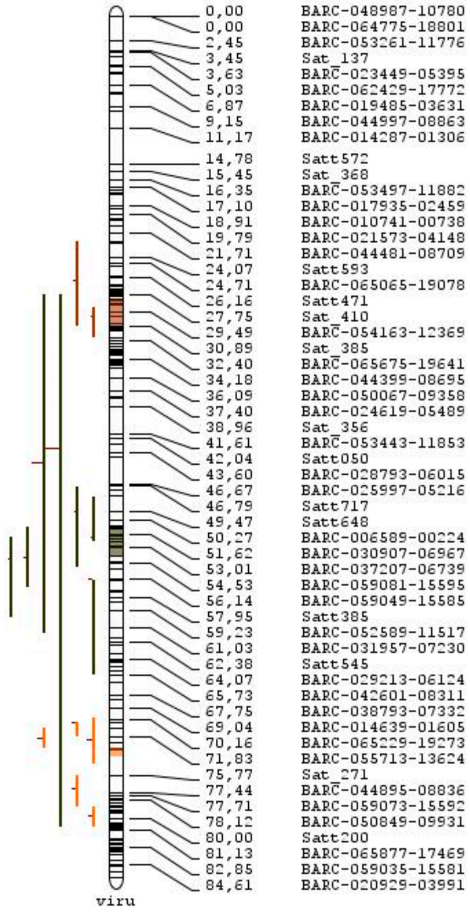
0,00 BARC-048987-10780
0,00 BARC-064775-18801
2,45 BARC-053261-11776
3,45 Sat_137
3,63 BARC-023449-05395
5,03 BARC-062429-17772
6,87 BARC-019485-03631
9,15 BARC-044997-08863
11,17 BARC-014287-01306
14,78 Satt572
15,45 Sat_368
16,35 BARC-053497-11882
17,10 BARC-017935-02459
18,91 BARC-010741-00738
19,79 BARC-021573-04148
21,71 BARC-044481-08709
24,07 Satt593
24,71 BARC-065065-19078
26,16 Satt471
27,75 Sat_410
29,49 BARC-054163-12369
30,89 Sat_385
32,40 BARC-065675-19641
34,18 BARC-044399-08695
36,09 BARC-050067-09358
37,40 BARC-024619-05489
38,96 Sat_356
41,61 BARC-053443-11853
42,04 Satt050
43,60 BARC-028793-06015
46,67 BARC-025997-05216
46,79 Satt717
49,47 Satt648
50,27 BARC-006589-00224
51,62 BARC-030907-06967
53,01 BARC-037207-06739
54,53 BARC-059081-15595
56,14 BARC-059049-15585
57,95 Satt385
59,23 BARC-052589-11517
61,03 BARC-031957-07230
62,38 Satt545
64,07 BARC-029213-06124
65,73 BARC-042601-08311
67,75 BARC-038793-07332
69,04 BARC-014639-01605
70,16 BARC-065229-19273
71,83 BARC-055713-13624
75,77 Sat_271
77,44 BARC-044895-08836
77,71 BARC-059073-15592
78,12 BARC-050849-09931
80,00 Satt200
81,13 BARC-065877-17469
82,85 BARC-059035-15581
84,61 BARC-020929-03991
viru

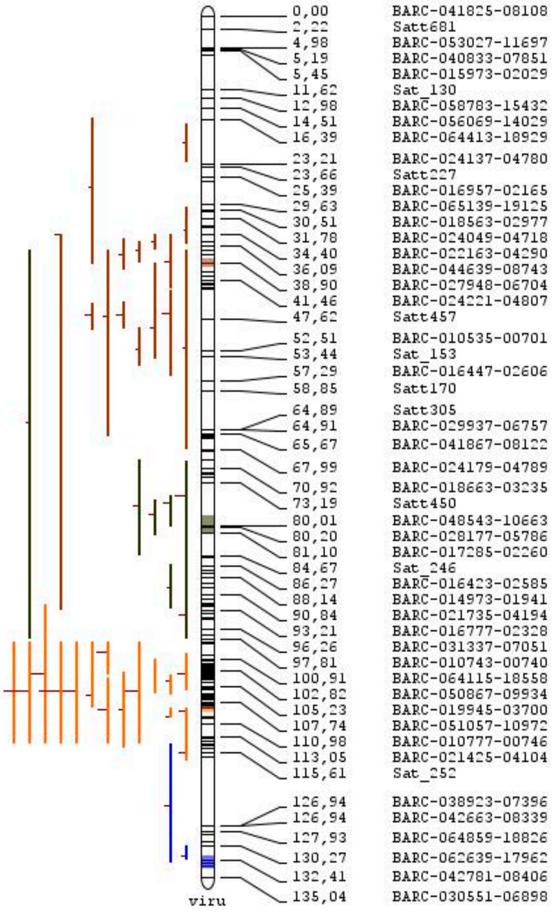
0,00 BARC-041825-08108
2,22 Satt681
4,98 BARC-053027-11697
5,19 BARC-040833-07851
5,45 BARC-015973-02029
11,62 Sat_130
12,98 BARC-058783-15432
14,51 BARC-056069-14029
16,39 BARC-064413-18929
23,21 BARC-024137-04780
23,66 Satt227
25,39 BARC-016957-02165
29,63 BARC-065139-19125
30,51 BARC-018563-02977
31,78 BARC-024049-04718
34,40 BARC-022163-04290
36,09 BARC-044639-08743
38,90 BARC-027948-06704
41,46 BARC-024221-04807
47,62 Satt457
52,51 BARC-010535-00701
53,44 Sat_153
57,29 BARC-016447-02606
58,85 Satt170
64,89 Satt305
64,91 BARC-029937-06757
65,67 BARC-041867-08122
67,99 BARC-024179-04789
70,92 BARC-018663-03235
73,19 Satt450
80,01 BARC-048543-10663
80,20 BARC-028177-05786
81,10 BARC-017285-02260
84,67 Sat_246
86,27 BARC-016423-02585
88,14 BARC-014973-01941
90,84 BARC-021735-04194
93,21 BARC-016777-02328
96,26 BARC-031337-07051
97,81 BARC-010743-00740
100,91 BARC-064115-18558
102,82 BARC-050867-09934
105,23 BARC-019945-03700
107,74 BARC-051057-10972
110,98 BARC-010777-00746
113,05 BARC-021425-04104
115,61 Sat_252
126,94 BARC-038923-07396
126,94 BARC-042663-08339
127,93 BARC-064859-18826
130,27 BARC-062639-17962
132,41 BARC-042781-08406
135,04 BARC-030551-06898
viru

6

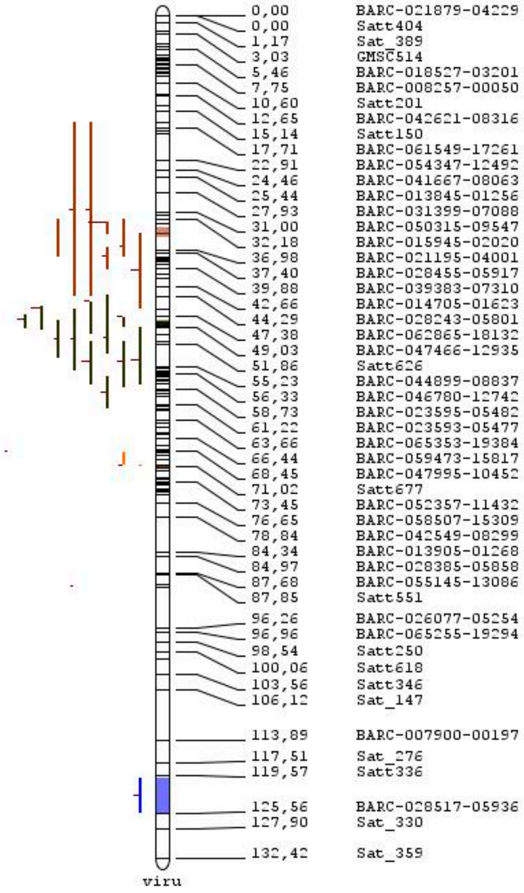
0,00 BARC-021879-04229
0,00 Satt404
1,17 Sat_389
3,03 GMSC514
5,46 BARC-018527-03201
7,75 BARC-008257-00050
10,60 Satt201
12,65 BARC-042621-08316
15,14 Satt150
17,71 BARC-061549-17261
22,91 BARC-054347-12492
24,46 BARC-041667-08063
25,44 BARC-013845-01256
27,93 BARC-031399-07088
31,00 BARC-050315-09547
32,18 BARC-015945-02020
36,98 BARC-021195-04001
37,40 BARC-028455-05917
39,88 BARC-039383-07310
42,66 BARC-014705-01623
44,29 BARC-028243-05801
47,38 BARC-062865-18132
49,03 BARC-047466-12935
51,86 Satt626
55,23 BARC-044899-08837
56,33 BARC-046780-12742
58,73 BARC-023595-05482
61,22 BARC-023593-05477
63,66 BARC-065353-19384
66,44 BARC-059473-15817
68,45 BARC-047995-10452
71,02 Satt677
73,45 BARC-052357-11432
76,65 BARC-058507-15309
78,84 BARC-042549-08299
84,34 BARC-013905-01268
84,97 BARC-028385-05858
87,68 BARC-055145-13086
87,85 Satt551
96,26 BARC-026077-05254
96,96 BARC-065255-19294
98,54 Satt250
100,06 Satt618
103,56 Satt346
106,12 Sat_147
113,89 BARC-007900-00197
117,51 Sat_276
119,57 Satt336
125,56 BARC-028517-05936
127,90 Sat_330
132,42 Sat_359
viru

7

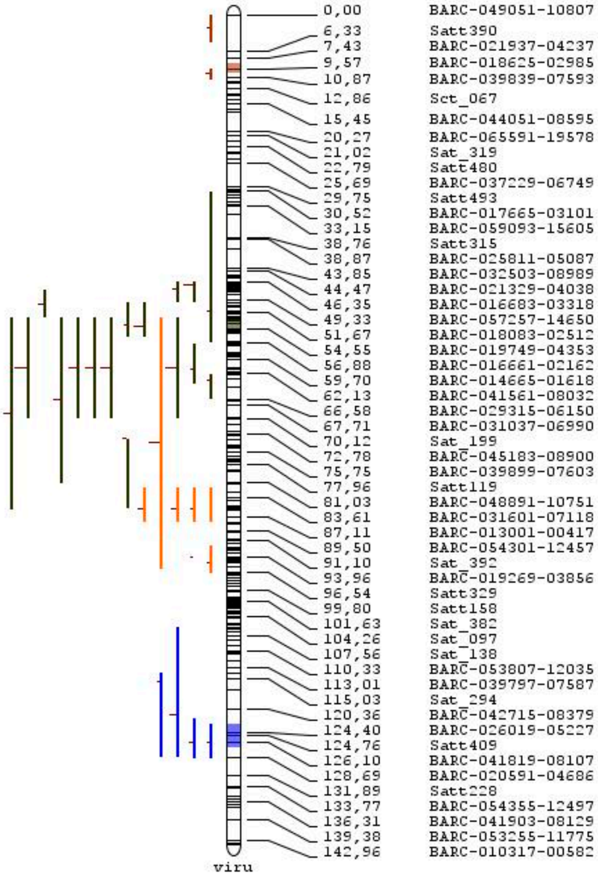
0,00 BARC-049051-10807
6,33 Satt390
7,43 BARC-021937-04237
9,57 BARC-018625-02985
10,87 BARC-039839-07593
12,86 Sct_067
15,45 BARC-044051-08595
20,27 BARC-065591-19578
21,02 Sat_319
22,79 Satt480
25,69 BARC-037229-06749
29,75 Satt493
30,52 BARC-017665-03101
33,15 BARC-059093-15605
38,76 Satt315
38,87 BARC-025811-05087
43,85 BARC-032503-08989
44,47 BARC-021329-04038
46,35 BARC-016683-03318
49,33 BARC-057257-14650
51,67 BARC-018083-02512
54,55 BARC-019749-04353
56,88 BARC-016661-02162
59,70 BARC-014665-01618
62,13 BARC-041561-08032
66,58 BARC-029315-06150
67,71 BARC-031037-06990
70,12 Sat_199
72,78 BARC-045183-08900
75,75 BARC-039899-07603
77,96 Satt119
81,03 BARC-048891-10751
83,61 BARC-031601-07118
87,11 BARC-013001-00417
89,50 BARC-054301-12457
91,10 Sat_392
93,96 BARC-019269-03856
96,54 Satt329
99,80 Satt158
101,63 Sat_382
104,26 Sat_097
107,56 Sat_138
110,33 BARC-053807-12035
113,01 BARC-039797-07587
115,03 Sat_294
120,36 BARC-042715-08379
124,40 BARC-026019-05227
124,76 Satt409
126,10 BARC-041819-08107
128,69 BARC-020591-04686
131,89 Satt228
133,77 BARC-054355-12497
136,31 BARC-041903-08129
139,38 BARC-053255-11775
142,96 BARC-010317-00582
viru

8

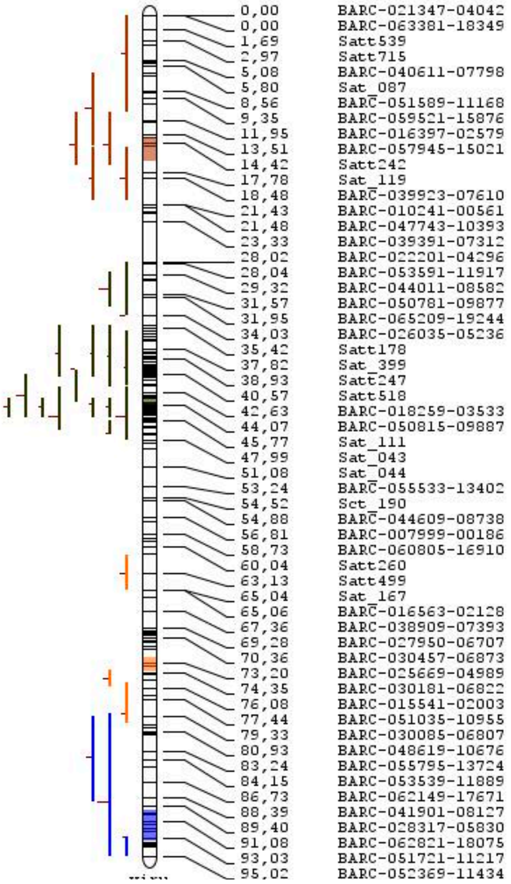

0,00 BARC-021347-04042
0,00 BARC-063381-18349
1,69 Satt539
2,97 Satt715
5,08 BARC-040611-07798
5,80 Sat_087
8,56 BARC-051589-11168
9,35 BARC-059521-15876
11,95 BARC-016397-02579
13,51 BARC-057945-15021
14,42 Satt242
17,78 Sat_119
18,48 BARC-039923-07610
21,43 BARC-010241-00561
21,48 BARC-047743-10393
23,33 BARC-039391-07312
28,02 BARC-022201-04296
28,04 BARC-053591-11917
29,32 BARC-044011-08582
31,57 BARC-050781-09877
31,95 BARC-065209-19244
34,03 BARC-026035-05236
35,42 Satt178
37,82 Sat_399
38,93 Satt247
40,57 Satt518
42,63 BARC-018259-03533
44,07 BARC-050815-09887
45,77 Sat_111
47,99 Sat_043
51,08 Sat_044
53,24 BARC-055533-13402
54,52 Sct_190
54,88 BARC-044609-08738
56,81 BARC-007999-00186
58,73 BARC-060805-16910
60,04 Satt260
63,13 Satt499
65,04 Sat_167
65,06 BARC-016563-02128
67,36 BARC-038909-07393
69,28 BARC-027950-06707
70,36 BARC-030457-06873
73,20 BARC-025669-04989
74,35 BARC-030181-06822
76,08 BARC-015541-02003
77,44 BARC-051035-10955
79,33 BARC-030085-06807
80,93 BARC-048619-10676
83,24 BARC-055795-13724
84,15 BARC-053539-11889
86,73 BARC-062149-17671
88,39 BARC-041901-08127
89,40 BARC-028317-05830
91,08 BARC-062821-18075
93,03 BARC-051721-11217
95,02 BARC-052369-11434

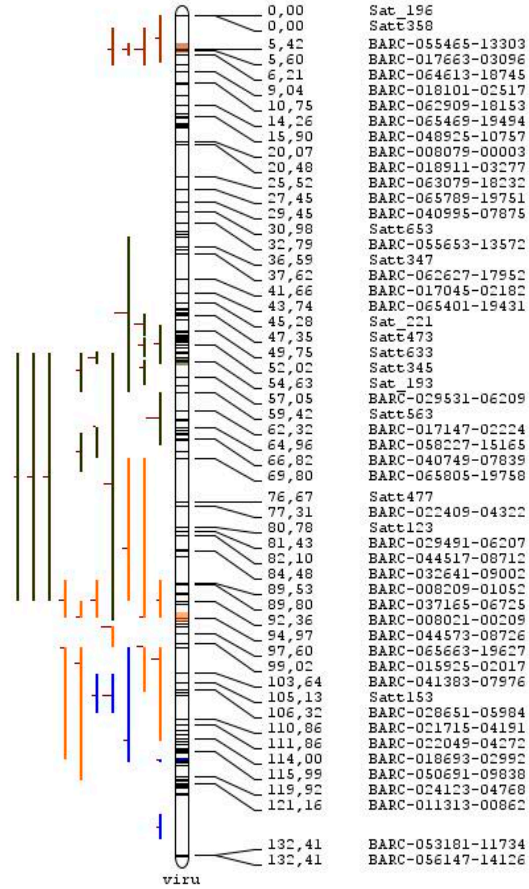

0,00 Sat_196
0,00 Satt358
5,42 BARC-055465-13303
5,60 BARC-017663-03096
6,21 BARC-064613-18745
9,04 BARC-018101-02517
10,75 BARC-062909-18153
14,26 BARC-065469-19494
15,90 BARC-048925-10757
20,07 BARC-008079-00003
20,48 BARC-018911-03277
25,52 BARC-063079-18232
27,45 BARC-065789-19751
29,45 BARC-040995-07875
30,98 Satt653
32,79 BARC-055653-13572
36,59 Satt347
37,62 BARC-062627-17952
41,66 BARC-017045-02182
43,74 BARC-065401-19431
45,28 Sat_221
47,35 Satt473
49,75 Satt633
52,02 Satt345
54,63 Sat_193
57,05 BARC-029531-06209
59,42 Satt563
62,32 BARC-017147-02224
64,96 BARC-058227-15165
66,82 BARC-040749-07839
69,80 BARC-065805-19758
76,67 Satt477
77,31 BARC-022409-04322
80,78 Satt123
81,43 BARC-029491-06207
82,10 BARC-044517-08712
84,48 BARC-032641-09002
89,53 BARC-008209-01052
89,80 BARC-037165-06725
92,36 BARC-008021-00209
94,97 BARC-044573-08726
97,60 BARC-065663-19627
99,02 BARC-015925-02017
103,64 BARC-041383-07976
105,13 Satt153
106,32 BARC-028651-05984
110,86 BARC-021715-04191
111,86 BARC-022049-04272
114,00 BARC-018693-02992
115,99 BARC-050691-09838
119,92 BARC-024123-04768
121,16 BARC-011313-00862
132,41 BARC-053181-11734
132,41 BARC-056147-14126
viru

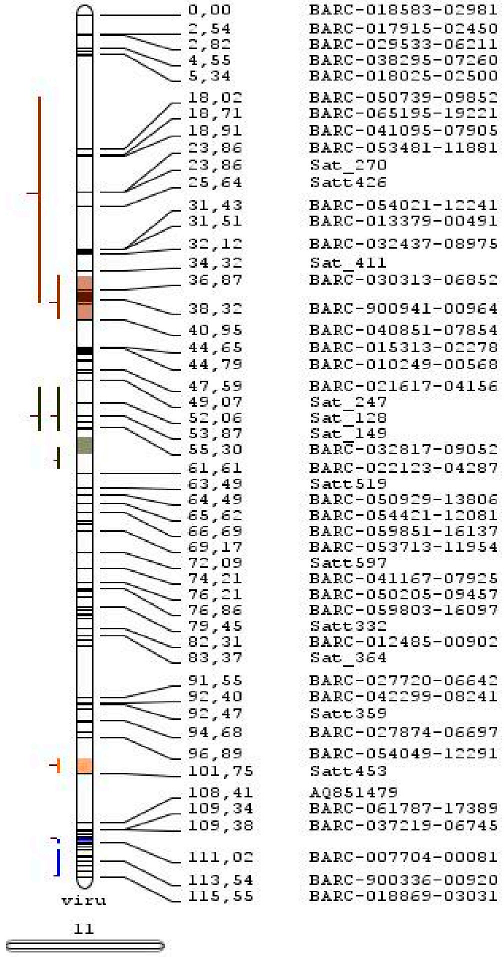

0,00 BARC-018583-02981
2,54 BARC-017915-02450
2,82 BARC-029533-06211
4,55 BARC-038295-07260
5,34 BARC-018025-02500
18,02 BARC-050739-09852
18,71 BARC-065195-19221
18,91 BARC-041095-07905
23,86 BARC-053481-11881
23,86 Sat_270
25,64 Satt426
31,43 BARC-054021-12241
31,51 BARC-013379-00491
32,12 BARC-032437-08975
34,32 Sat_411
36,87 BARC-030313-06852
38,32 BARC-900941-00964
40,95 BARC-040851-07854
44,65 BARC-015313-02278
44,79 BARC-010249-00568
47,59 BARC-021617-04156
49,07 Sat_247
52,06 Sat_128
53,87 Sat_149
55,30 BARC-032817-09052
61,61 BARC-022123-04287
63,49 Satt519
64,49 BARC-050929-13806
65,62 BARC-054421-12081
66,69 BARC-059851-16137
69,17 BARC-053713-11954
72,09 Satt597
74,21 BARC-041167-07925
76,21 BARC-050205-09457
76,86 BARC-059803-16097
79,45 Satt332
82,31 BARC-012485-00902
83,37 Sat_364
91,55 BARC-027720-06642
92,40 BARC-042299-08241
92,47 Satt359
94,68 BARC-027874-06697
96,89 BARC-054049-12291
101,75 Satt453
108,41 AQ851479
109,34 BARC-061787-17389
109,38 BARC-037219-06745
111,02 BARC-007704-00081
113,54 BARC-900336-00920
115,55 BARC-018869-03031
viru
11

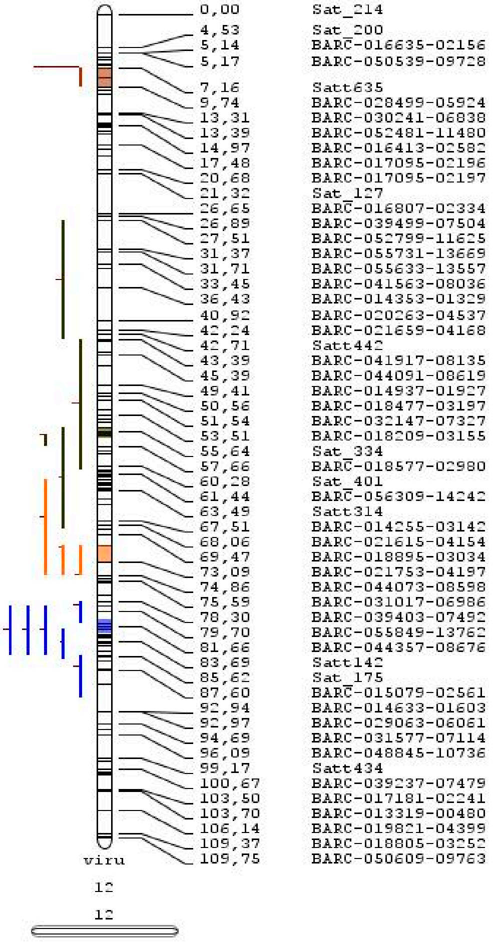

0,00 Sat_214
4,53 Sat_200
5,14 BARC-016635-02156
5,17 BARC-050539-09728
7,16 Satt635
9,74 BARC-028499-05924
13,31 BARC-030241-06838
13,39 BARC-052481-11480
14,97 BARC-016413-02582
17,48 BARC-017095-02196
20,68 BARC-017095-02197
21,32 Sat_127
26,65 BARC-016807-02334
26,89 BARC-039499-07504
27,51 BARC-052799-11625
31,37 BARC-055731-13669
31,71 BARC-055633-13557
33,45 BARC-041563-08036
36,43 BARC-014353-01329
40,92 BARC-020263-04537
42,24 BARC-021659-04168
42,71 Satt442
43,39 BARC-041917-08135
45,39 BARC-044091-08619
49,41 BARC-014937-01927
50,56 BARC-018477-03197
51,54 BARC-032147-07327
53,51 BARC-018209-03155
55,64 Sat_334
57,66 BARC-018577-02980
60,28 Sat_401
61,44 BARC-056309-14242
63,49 Satt314
67,51 BARC-014255-03142
68,06 BARC-021615-04154
69,47 BARC-018895-03034
73,09 BARC-021753-04197
74,86 BARC-044073-08598
75,59 BARC-031017-06986
78,30 BARC-039403-07492
79,70 BARC-055849-13762
81,66 BARC-044357-08676
83,69 Satt142
85,62 Sat_175
87,60 BARC-015079-02561
92,94 BARC-014633-01603
92,97 BARC-029063-06061
94,69 BARC-031577-07114
96,09 BARC-048845-10736
99,17 Satt434
100,67 BARC-039237-07479
103,50 BARC-017181-02241
103,70 BARC-013319-00480
106,14 BARC-019821-04399
109,37 BARC-018805-03252
109,75 BARC-050609-09763
viru
12
12

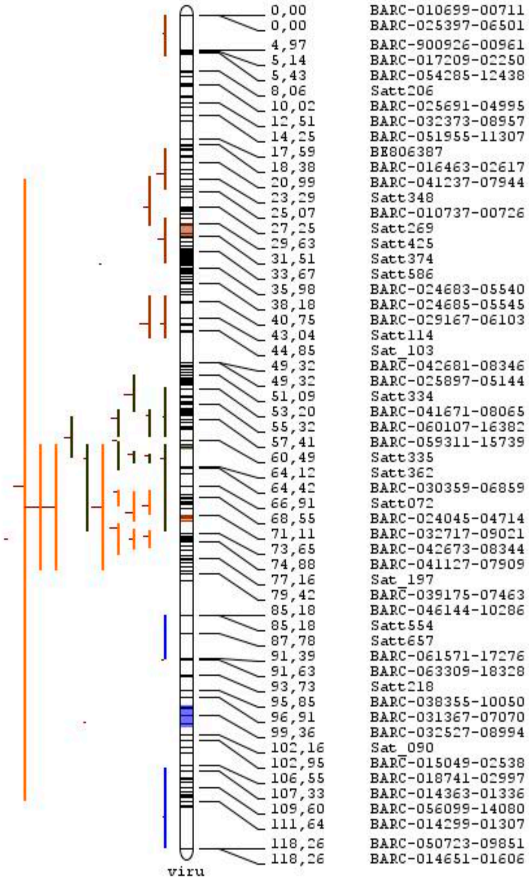
0,00 BARC-010699-00711
0,00 BARC-025397-06501
4,97 BARC-900926-00961
5,14 BARC-017209-02250
5,43 BARC-054285-12438
8,06 Satt206
10,02 BARC-025691-04995
12,51 BARC-032373-08957
14,25 BARC-051955-11307
17,59 BE806387
18,38 BARC-016463-02617
20,99 BARC-041237-07944
23,29 Satt348
25,07 BARC-010737-00726
27,25 Satt269
29,63 Satt425
31,51 Satt374
33,67 Satt586
35,98 BARC-024683-05540
38,18 BARC-024685-05545
40,75 BARC-029167-06103
43,04 Satt114
44,85 Sat_103
49,32 BARC-042681-08346
49,32 BARC-025897-05144
51,09 Satt334
53,20 BARC-041671-08065
55,32 BARC-060107-16382
57,41 BARC-059311-15739
60,49 Satt335
64,12 Satt362
64,42 BARC-030359-06859
66,91 Satt072
68,55 BARC-024045-04714
71,11 BARC-032717-09021
73,65 BARC-042673-08344
74,88 BARC-041127-07909
77,16 Sat_197
79,42 BARC-039175-07463
85,18 BARC-046144-10286
85,18 Satt554
87,78 Satt657
91,39 BARC-061571-17276
91,63 BARC-063309-18328
93,73 Satt218
95,85 BARC-038355-10050
96,91 BARC-031367-07070
99,36 BARC-032527-08994
102,16 Sat_090
102,95 BARC-015049-02538
106,55 BARC-018741-02997
107,33 BARC-014363-01336
109,60 BARC-056099-14080
111,64 BARC-014299-01307
118,26 BARC-050723-09851
118,26 BARC-014651-01606
viru

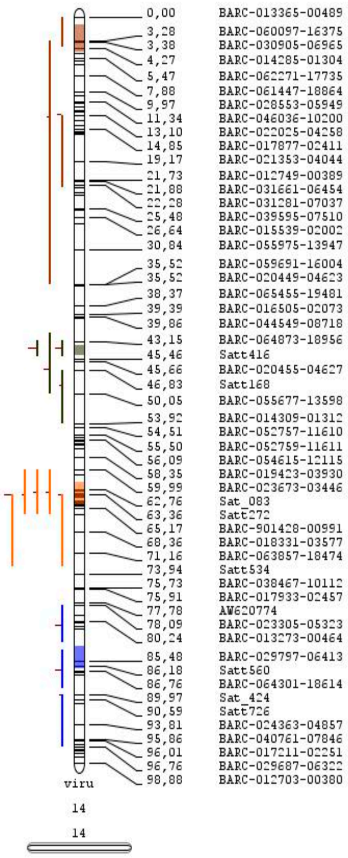
0,00 BARC-013365-00489
3,28 BARC-060097-16375
3,38 BARC-030905-06965
4,27 BARC-014285-01304
5,47 BARC-062271-17735
7,88 BARC-061447-18864
9,97 BARC-028553-05949
11,34 BARC-046036-10200
13,10 BARC-022025-04258
14,85 BARC-017877-02411
19,17 BARC-021353-04044
21,73 BARC-012749-00389
21,88 BARC-031661-06454
22,28 BARC-031281-07037
25,48 BARC-039595-07510
26,64 BARC-015539-02002
30,84 BARC-055975-13947
35,52 BARC-059691-16004
35,52 BARC-020449-04623
38,37 BARC-065455-19481
39,39 BARC-016505-02073
39,86 BARC-044549-08718
43,15 BARC-064873-18956
45,46 Satt416
45,66 BARC-020455-04627
46,83 Satt168
50,05 BARC-055677-13598
53,92 BARC-014309-01312
54,51 BARC-052757-11610
55,50 BARC-052759-11611
56,09 BARC-054615-12115
58,35 BARC-019423-03930
59,99 BARC-023673-03446
62,76 Sat_083
63,36 Satt272
65,17 BARC-901428-00991
68,36 BARC-018331-03577
71,16 BARC-063857-18474
73,94 Satt534
75,73 BARC-038467-10112
75,91 BARC-017933-02457
77,78 AW620774
78,09 BARC-023305-05323
80,24 BARC-013273-00464
85,48 BARC-029797-06413
86,18 Satt560
86,76 BARC-064301-18614
89,97 Sat_424
90,59 Satt726
93,81 BARC-024363-04857
95,86 BARC-040761-07846
96,01 BARC-017211-02251
96,76 BARC-029687-06322
98,88 BARC-012703-00380
viru
14
14

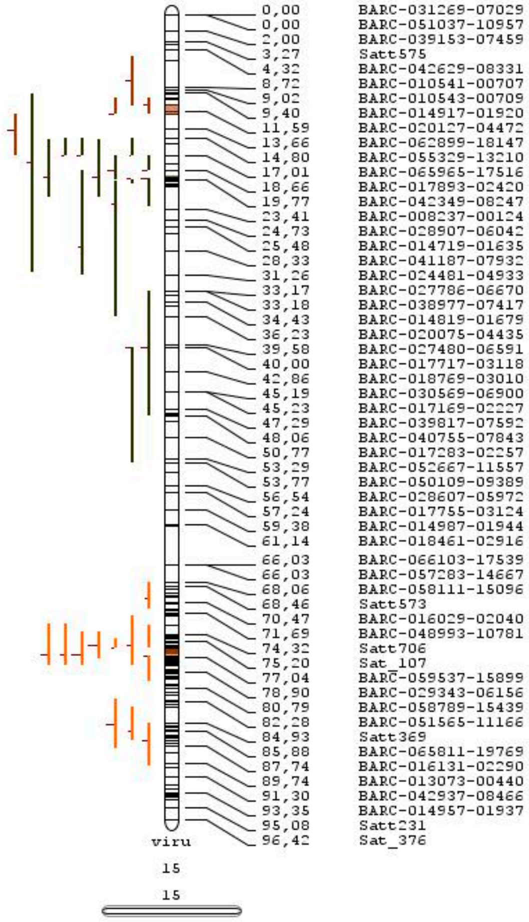
0,00 BARC-031269-07029
0,00 BARC-051037-10957
2,00 BARC-039153-07459
3,27 Satt575
4,32 BARC-042629-08331
8,72 BARC-010541-00707
9,02 BARC-010543-00709
9,40 BARC-014917-01920
11,59 BARC-020127-04472
13,66 BARC-062899-18147
14,80 BARC-055329-13210
17,01 BARC-065965-17516
18,66 BARC-017893-02420
19,77 BARC-042349-08247
23,41 BARC-008237-00124
24,73 BARC-028907-06042
25,48 BARC-014719-01635
28,33 BARC-041187-07932
31,26 BARC-024481-04933
33,17 BARC-027786-06670
33,18 BARC-038977-07417
34,43 BARC-014819-01679
36,23 BARC-020075-04435
39,58 BARC-027480-06591
40,00 BARC-017717-03118
42,86 BARC-018769-03010
45,19 BARC-030569-06900
45,23 BARC-017169-02227
47,29 BARC-039817-07592
48,06 BARC-040755-07843
50,77 BARC-017283-02257
53,29 BARC-052667-11557
53,77 BARC-050109-09389
56,54 BARC-028607-05972
57,24 BARC-017755-03124
59,38 BARC-014987-01944
61,14 BARC-018461-02916
66,03 BARC-066103-17539
66,03 BARC-057283-14667
68,06 BARC-058111-15096
68,46 Satt573
70,47 BARC-016029-02040
71,69 BARC-048993-10781
74,32 Satt706
75,20 Sat_107
77,04 BARC-059537-15899
78,90 BARC-029343-06156
80,79 BARC-058789-15439
82,28 BARC-051565-11166
84,93 Satt369
85,88 BARC-065811-19769
87,74 BARC-016131-02290
89,74 BARC-013073-00440
91,30 BARC-042937-08466
93,35 BARC-014957-01937
95,08 Satt231
96,42 Sat_376
viru
15
15

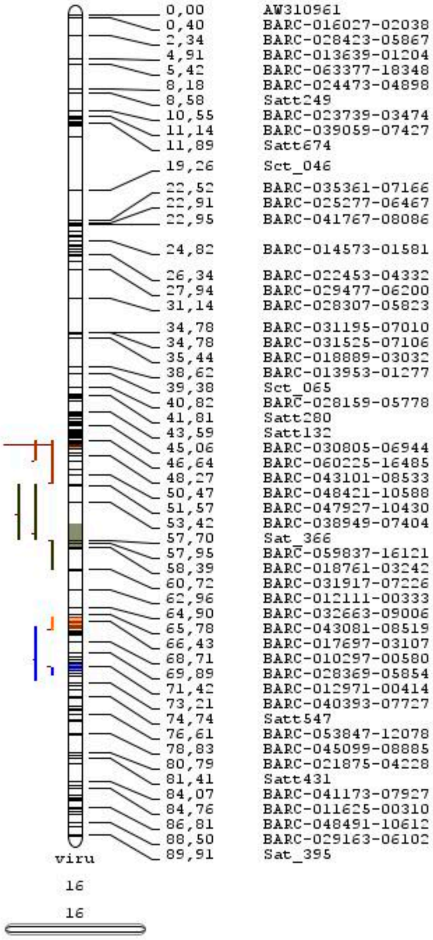
0,00 AW310961
0,40 BARC-016027-02038
2,34 BARC-028423-05867
4,91 BARC-013639-01204
5,42 BARC-063377-18348
8,18 BARC-024473-04898
8,58 Satt249
10,55 BARC-023739-03474
11,14 BARC-039059-07427
11,89 Satt674
19,26 Sct_046
22,52 BARC-035361-07166
22,91 BARC-025277-06467
22,95 BARC-041767-08086
24,82 BARC-014573-01581
26,34 BARC-022453-04332
27,94 BARC-029477-06200
31,14 BARC-028307-05823
34,78 BARC-031195-07010
34,78 BARC-031525-07106
35,44 BARC-018889-03032
38,62 BARC-013953-01277
39,38 Sct_065
40,82 BARC-028159-05778
41,81 Satt280
43,59 Satt132
45,06 BARC-030805-06944
46,64 BARC-060225-16485
48,27 BARC-043101-08533
50,47 BARC-048421-10588
51,57 BARC-047927-10430
53,42 BARC-038949-07404
57,70 Sat_366
57,95 BARC-059837-16121
58,39 BARC-018761-03242
60,72 BARC-031917-07226
62,96 BARC-012111-00333
64,90 BARC-032663-09006
65,78 BARC-043081-08519
66,43 BARC-017697-03107
68,71 BARC-010297-00580
69,89 BARC-028369-05854
71,42 BARC-012971-00414
73,21 BARC-040393-07727
74,74 Satt547
76,61 BARC-053847-12078
78,83 BARC-045099-08885
80,79 BARC-021875-04228
81,41 Satt431
84,07 BARC-041173-07927
84,76 BARC-011625-00310
86,81 BARC-048491-10612
88,50 BARC-029163-06102
89,91 Sat_395
viru
16
16

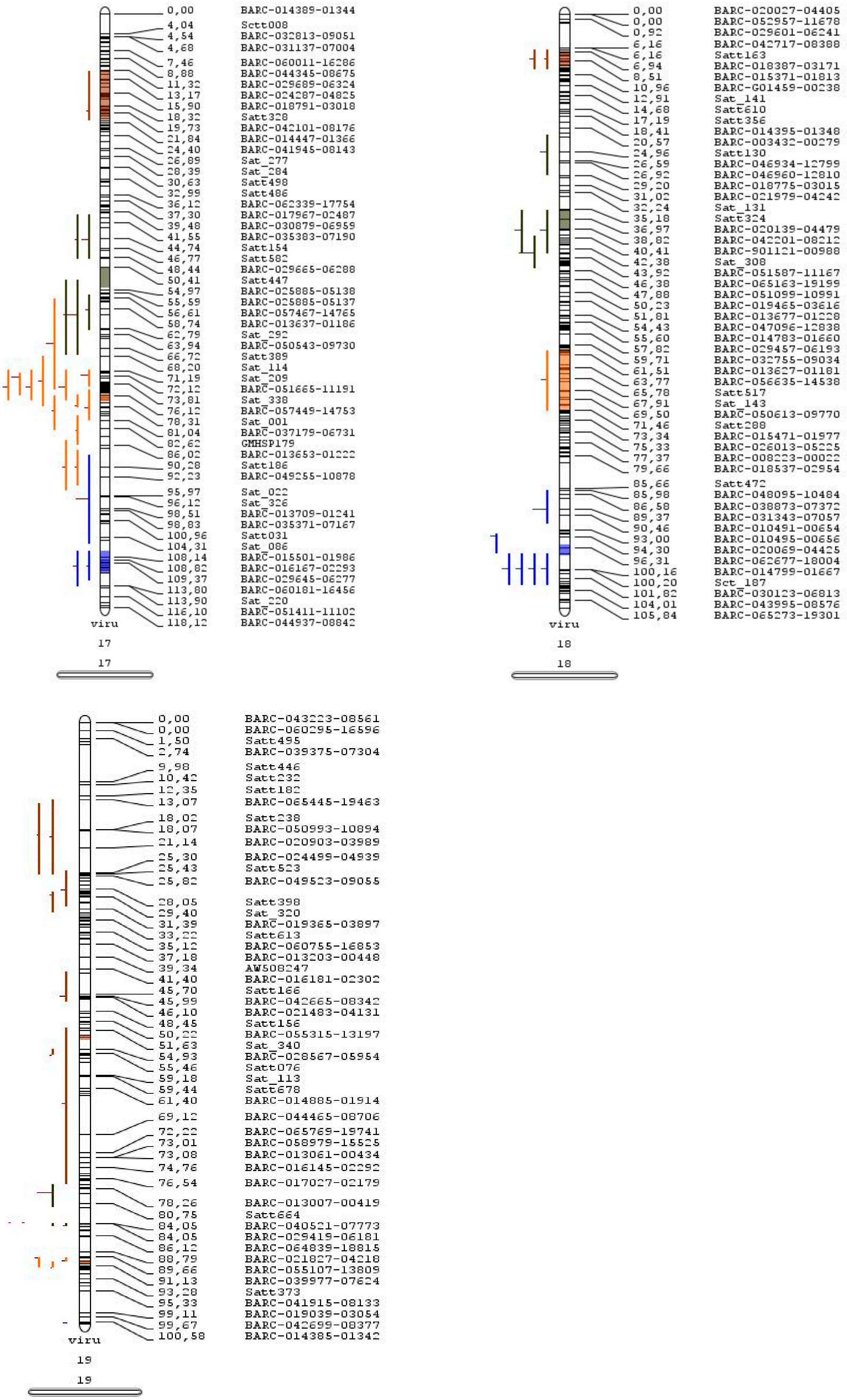
0,00 BARC-014389-01344
4,04 Sctt008
4,54 BARC-032813-09051
4,68 BARC-031137-07004
7,46 BARC-060011-16286
8,88 BARC-044345-08675
11,32 BARC-029689-06324
13,17 BARC-024287-04825
15,90 BARC-018791-03018
18,32 Satt328
19,73 BARC-042101-08176
21,84 BARC-014447-01366
24,40 BARC-041945-08143
26,89 Sat_277
28,39 Sat_284
30,63 Satt498
32,99 Satt486
36,12 BARC-062339-17754
37,30 BARC-017967-02487
39,48 BARC-030879-06959
41,55 BARC-035383-07190
44,74 Satt154
46,77 Satt582
48,44 BARC-029665-06288
50,41 Satt447
54,97 BARC-025885-05138
55,59 BARC-025885-05137
56,61 BARC-057467-14765
58,74 BARC-013637-01186
62,79 Sat_292
63,94 BARC-050543-09730
66,72 Satt389
68,20 Sat_114
71,19 Sat_209
72,12 BARC-051665-11191
73,81 Sat_338
76,12 BARC-057449-14753
78,31 Sat_001
81,04 BARC-037179-06731
82,62 GMHSP179
86,02 BARC-013653-01222
90,28 Satt186
92,23 BARC-049255-10878
95,97 Sat_022
96,12 Sat_326
98,51 BARC-013709-01241
98,83 BARC-035371-07167
100,96 Satt031
104,31 Sat_086
108,14 BARC-015501-01986
108,82 BARC-016167-02293
109,37 BARC-029645-06277
113,80 BARC-060181-16456
113,90 Sat_220
116,10 BARC-051411-11102
118,12 BARC-044937-08842
viru
17
17
0,00 BARC-020027-04405
0,00 BARC-052957-11678
0,92 BARC-029601-06241
6,16 BARC-042717-08388
6,16 Satt163
6,94 BARC-018387-03171
8,51 BARC-015371-01813
10,96 BARC-G01459-00238
12,91 Sat_141
14,68 Satt610
17,19 Satt356
18,41 BARC-014395-01348
20,57 BARC-003432-00279
24,96 Satt130
26,59 BARC-046934-12799
26,92 BARC-046960-12810
29,20 BARC-018775-03015
31,02 BARC-021979-04242
32,24 Sat_131
35,18 Satt324
36,97 BARC-020139-04479
38,82 BARC-042201-08212
40,41 BARC-901121-00988
42,38 Sat_308
43,92 BARC-051587-11167
46,38 BARC-065163-19199
47,88 BARC-051099-10991
50,23 BARC-019465-03616
51,81 BARC-013677-01228
54,43 BARC-047096-12838
55,60 BARC-014783-01660
57,82 BARC-029457-06193
59,71 BARC-032755-09034
61,51 BARC-013627-01181
63,77 BARC-056635-14538
65,78 Satt517
67,91 Sat_143
69,50 BARC-050613-09770
71,46 Satt288
73,34 BARC-015471-01977
75,33 BARC-026013-05225
77,37 BARC-008223-00022
79,66 BARC-018537-02954
85,66 Satt472
85,98 BARC-048095-10484
86,58 BARC-038873-07372
89,37 BARC-031343-07057
90,46 BARC-010491-00654
93,00 BARC-010495-00656
94,30 BARC-020069-04425
96,31 BARC-062677-18004
100,16 BARC-014799-01667
100,20 Sct_187
101,82 BARC-030123-06813
104,01 BARC-043995-08576
105,84 BARC-065273-19301
viru
18
18
0,00 BARC-043223-08561
0,00 BARC-060295-16596
1,50 Satt495
2,74 BARC-039375-07304
9,98 Satt446
10,42 Satt232
12,35 Satt182
13,07 BARC-065445-19463
18,02 Satt238
18,07 BARC-050993-10894
21,14 BARC-020903-03989
25,30 BARC-024499-04939
25,43 Satt523
25,82 BARC-049523-09055
28,05 Satt398
29,40 Sat_320
31,39 BARC-019365-03897
33,22 Satt613
35,12 BARC-060755-16853
37,18 BARC-013203-00448
39,34 AW508247
41,40 BARC-016181-02302
45,70 Satt166
45,99 BARC-042665-08342
46,10 BARC-021483-04131
48,45 Satt156
50,22 BARC-055315-13197
51,63 Sat_340
54,93 BARC-028567-05954
55,46 Satt076
59,18 Sat_113
59,44 Satt678
61,40 BARC-014885-01914
69,12 BARC-044465-08706
72,22 BARC-065769-19741
73,01 BARC-058979-15525
73,08 BARC-013061-00434
74,76 BARC-016145-02292
76,54 BARC-017027-02179
78,26 BARC-013007-00419
80,75 Satt664
84,05 BARC-040521-07773
84,05 BARC-029419-06181
86,12 BARC-064839-18815
88,79 BARC-021827-04218
89,66 BARC-055107-13809
91,13 BARC-039977-07624
93,28 Satt373
95,33 BARC-041915-08133
99,11 BARC-019039-03054
99,67 BARC-042699-08377
100,58 BARC-014385-01342
viru
19
19
