# Supplementary material for: Pinpointing Genomic Regions and Candidate Genes Associated with Seed Oil and Protein Content in Soybean through an Integrative Transcriptomic and QTL Meta-Analysis
Source: Cells. 2022 Dec 26;12(1):97. doi: 10.3390/cells12010097 (PMC9818467; doi:10.3390/cells12010097)
Supplement: Supplementary file 1 [file cells-12-00097-s001.zip › Table S 1.pdf]

Table S1b. List of quantitative trait loci (QTLs) reported for seed oil and protein content in different studies used for meta-QTL analysis in soybean.

Study	Population Size	Supplementary Figures.pdf<br>Crosses	Population Type	Mapping Method
Akond, *et al.* [1]	50	PI 438489B and Hamilton	F6:13	CIM
Akond, *et al.* [2]	92	MD96-5722 x Spencer	F5:7	CIM
Asekova, *et al.* [3]	188	PI 483463 and Hutcheson	F5:8	CIM
Brummer, *et al.* [4]	70 to 100	Multiple (eight)	F2:4	ANOVA
Chapman, *et al.* [5]	177	Essex' and 'Williams	F2:4	ANOVA
Chen, *et al.* [6]	154	Charleston x Dongnong594	RIL	CIM
Chung, *et al.* [7]	76	Asgrow A3733 x PI 437088A	F5:6	SIM, CIM
Csanádi, *et al.* [8]	82	Ma.Belle x Proto	F2	CIM
Hyten, *et al.* [9]	131	Essex' and 'Williams	F6	CIM
Gai, *et al.* [10]	201	Kefeng No.1 x 1138-2	F2:7:10	CIM
Kabelka, *et al.* [11]	167	BSR 101 x LG82-8379	F5:8, F5:9	CIM
Lee, *et al.* [12]	120 and 111	Young x PI 416 937; and PI **97** 100 x "Coker 237	F2	IM
Liang, *et al.* [13]	474	Jindou 23 and Huibuzhi	F13	CIM
Mansur, *et al.* [14]		Minsoy x 'Noir 1	F2	IM
Mao, *et al.* [15]	148 and 109	HeFeng 47 x HeiNong 37; and HeFeng 47 x HeiNong 44	F5:6	CIM
Eskandari, *et al.* [16]	203	OAC Wallace x OAC Glencoe	F4:6	SIM, CIM
Moongkanna, *et al.* [17]	186	Pak Chong 2 and Laos 7122	F2:3	CIM
Orf, *et al.* [18]	240 and 233	Minsoy x 'Noir 1; and Archer x Minsoy	F7	IM
Panthee, *et al.* [19]	101	N87-984-16 x TN93-99	F6	CIM
Parmley [20]	118	PI479762XPI567214B	F4	ICMI
Pathan, *et al.* [21]	216 and 156	Magellan x PI 438489B; and Magellan x PI 567516C	F5:6; F5:7	MQM
Patil, *et al.* [22]	188	Williams 82 x PI 483460B	RIL	CIM
Phansak, *et al.* [23]	250	48 different population	F2	IM

Commented [M1]: Please check if this should be a multiplication sign ("×" U+00D7).Same below.

Commented [DH2R1]: "x" is used as a symbol for a genetic cross. This is well-accepted practice in plant breeding

Commented [M3]: If the line is not necessary. Please remove it.

Commented [DH4R3]: Line removed

Commented [AW5]: Please ensure intended meaning is retained.

Commented [DH6R5]: yes

Supplementary Figures.pdf

Study	Population Size	Crosses	Population Type	Mapping Method
Qi, *et al.* [24]	154	Charleston x Dongnong594	RIL	CIM
Qi, *et al.* [25]	147	Charleston x Dongnong594	F2:18	CIM & MIM
Zhaoming, *et al.* [26]	147	Charleston x Dongnong594	F2:18	CIM
Qiu, *et al.* [27]	200	Peking x Essex	F2:3	CIM
Reinprecht, *et al.* [28]	169	RG10 x OX948	F5	CIM
Seo, *et al.* [29]	220	Neulchan x 'Saedanbaek	F2:10	ICIM
Shibata, *et al.* [30]	96	TK 780 and Hidaka 4	RIL	IM
Smallwood, *et al.* [31]	203	Essex and Williams 82	F5	IM
Tajuddin, *et al.* [32]	156	Misuzudaizu x Moshidou Gong 503	F8	CIM
Wang, *et al.* [33]	87	SD02-4-59 9 A02-381100	F5	IM, CIM
Wang, *et al.* [34]	87	SD02-4-59 9 A02-381100	F5	IM, CIM
Wang, *et al.* [35]	242 and 214	R05-1415 xR05- 638; V97-1346 x R05-4256	F2:3	CIM
Li, *et al.* [36]	118	PI479762 and PI567214B	RIL	ICMI
Zhang, *et al.* [37]	184	Kefeng No.1 x Nannong 1138-2	F2:7	CIM
Leite, *et al.* [38]	244	Lineage 69 x Tucunaré	F2:3	CIM

Commented [M7]: Please change the citation into [X]/[X,X]/[X–X]

1. Akond, A.M.; Ragin, B.; Bazzelle, R.; Kantartzi, S.K.; Meksem, K.; Kassem, M.A. Quantitative trait loci associated with moisture, protein, and oil content in soybean [Glycine max (L.) Merr.]. *Journal of Agricultural Science* **2012**, *4*, 16.
2. Akond, M.; Liu, S.; Boney, M.; Kantartzi, S.K.; Meksem, K.; Bellaloui, N.; Lightfoot, D.A.; Kassem, M.A. Identification of quantitative trait loci (QTL) underlying protein, oil, and five major fatty acids' contents in soybean. *American Journal of Plant Sciences* **2014**, *2014*.
3. Asekova, S.; Kulkarni, K.P.; Kim, M.; Kim, J.H.; Song, J.T.; Shannon, J.G.; Lee, J.D. Novel quantitative trait loci for forage quality traits in a cross between PI 483463 and 'Hutcheson'in soybean. *Crop Science* **2016**, *56*, 2600-2611.
4. Brummer, E.C.; Graef, G.L.; Orf, J.; Wilcox, J.R.; Shoemaker, R.C. Mapping QTL for seed protein and oil content in eight soybean populations. *Crop Science* **1997**, *37*, 370-378.
5. Chapman, A.; Pantalone, V.R.; Ustun, A.; Allen, F.L.; Landau-Ellis, D.; Trigiano, R.N.; Gresshoff, P.M. Quantitative trait loci for agronomic and seed quality traits in an F2 and F4: 6 soybean population. *Euphytica* **2003**, *129*, 387-393.

6. Chen, Q.-S.; Zhang, Z.-C.; Liu, C.-Y.; Xin, D.-W.; Qiu, H.-M.; Shan, D.-P.; Shan, C.-Y.; Hu, G.-H. QTL analysis of major agronomic traits in soybean. *Agricultural Sciences in China* **2007**, *6*, 399-405.
7. Chung, J.; Babka, H.; Graef, G.; Staswick, P.; Lee, D.; Cregan, P.; Shoemaker, R.; Specht, J.J.C.s. The seed protein, oil, and yield QTL on soybean linkage group I. **2003**, *43*, 1053-1067.
8. Csanádi, G.Y.; Vollmann, J.; Stift, G.; Lelley, T. Seed quality QTLs identified in a molecular map of early maturing soybean. *Theoretical and Applied Genetics* **2001**, *103*, 912-919.
9. Hyten, D.L.; Pantalone, V.R.; Sams, C.E.; Saxton, A.M.; Landau-Ellis, D.; Stefaniak, T.R.; Schmidt, M.E. Seed quality QTL in a prominent soybean population. *Theoretical and Applied Genetics* **2004**, *109*, 552-561.
10. Gai, J.; Wang, Y.; Wu, X.; Chen, S. A comparative study on segregation analysis and QTL mapping of quantitative traits in plants—with a case in soybean. *Frontiers of Agriculture in China* **2007**, *1*, 1-7.
11. Kabelka, E.A.; Diers, B.W.; Fehr, W.R.; LeRoy, A.R.; Baianu, I.C.; You, T.; Neece, D.J.; Nelson, R.L. Putative alleles for increased yield from soybean plant introductions. *Crop science* **2004**, *44*, 784-791.
12. Lee, S.H.; Bailey, M.A.; Mian, M.A.R.; Carter, T.E.; Shipe, E.R.; Ashley, D.A.; Parrott, W.A.; Hussey, R.S.; Boerma, H.R. RFLP loci associated with soybean seed protein and oil content across populations and locations. *Theoretical and Applied Genetics* **1996**, *93*, 649-657.
13. Liang, H.-Z.; Yu, Y.-L.; Wang, S.-F.; Yun, L.; Wang, T.-F.; Wei, Y.-L.; Gong, P.-T.; Liu, X.-Y.; Fang, X.-J.; Zhang, M.-C. QTL mapping of isoflavone, oil and protein contents in soybean (Glycine max L. Merr.). *Agricultural Sciences in China* **2010**, *9*, 1108-1116.
14. Mansur, L.M.; Lark, K.G.; Kross, H.; Oliveira, A. Interval mapping of quantitative trait loci for reproductive, morphological, and seed traits of soybean (Glycine max L.). *Theoretical and Applied Genetics* **1993**, *86*, 907-913.
15. Mao, T.; Jiang, Z.; Han, Y.; Teng, W.; Zhao, X.; Li, W. Identification of quantitative trait loci underlying seed protein and oil contents of soybean across multi-genetic backgrounds and environments. *Plant Breeding* **2013**, *132*, 630-641.
16. Eskandari, M.; Cober, E.R.; Rajcan, I. Genetic control of soybean seed oil: I. QTL and genes associated with seed oil concentration in RIL populations derived from crossing moderately high-oil parents. *Theoretical and Applied Genetics* **2013**, *126*, 483-495.
17. Moongkanna, J.; Nakasathien, S.; Novitzky, W.P.; Kwanyuen, P.; Sinchaisri, P.; Srinives, P. SSR markers linking to seed traits and total oil content in soybean. *Thai Journal of Agricultural Science* **2011**, *44*, 233-241.
18. Orf, J.H.; Chase, K.; Jarvik, T.; Mansur, L.M.; Cregan, P.B.; Adler, F.R.; Lark, K.G. Genetics of soybean agronomic traits: I. Comparison of three related recombinant inbred populations. *Crop Science* **1999**, *39*, 1642-1651.
19. Panthee, D.R.; Pantalone, V.R.; West, D.R.; Saxton, A.M.; Sams, C.E. Quantitative trait loci for seed protein and oil concentration, and seed size in soybean. *Crop Science* **2005**, *45*, 2015-2022.
20. Parmley, K. Quantitative trail loci mapping of seed protein and oil composition in a diverse soybean recombinant inbred line population. **2016**.
21. Pathan, S.M.; Vuong, T.; Clark, K.; Lee, J.D.; Shannon, J.G.; Roberts, C.A.; Ellersieck, M.R.; Burton, J.W.; Cregan, P.B.; Hyten, D.L. Genetic mapping and confirmation of quantitative trait loci for seed protein and oil contents and seed weight in soybean. *Crop Science* **2013**, *53*, 765-774.
22. Patil, G.; Vuong, T.D.; Kale, S.; Valliyodan, B.; Deshmukh, R.; Zhu, C.; Wu, X.; Bai, Y.; Yungbluth, D.; Lu, F. Dissecting genomic hotspots underlying seed protein, oil, and sucrose content in an interspecific mapping population of soybean using high-density linkage mapping. *Plant Biotechnology Journal* **2018**, *16*, 1939-1953.

23. Phansak, P.; Soonsuwon, W.; Hyten, D.L.; Song, Q.; Cregan, P.B.; Graef, G.L.; Specht, J.E. Multi-population selective genotyping to identify soybean [Glycine max (L.) Merr.] seed protein and oil QTLs. *G3: Genes, Genomes, Genetics* **2016**, *6*, 1635-1648.
24. Qi, Z.-m.; Wu, Q.; Han, X.; Sun, Y.-n.; Du, X.-y.; Liu, C.-y.; Jiang, H.-w.; Hu, G.-h.; Chen, Q.-s. Soybean oil content QTL mapping and integrating with meta-analysis method for mining genes. *Euphytica* **2011**, *179*, 499-514.
25. Qi, Z.; Hou, M.; Han, X.; Liu, C.; Jiang, H.; Xin, D.; Hu, G.; Chen, Q. Identification of quantitative trait loci (QTL s) for seed protein concentration in soybean and analysis for additive effects and epistatic effects of QTL s under multiple environments. *Plant Breeding* **2014**, *133*, 499-507.
26. Zhaoming, Q.; Xiaoying, Z.; Huidong, Q.; Dawei, X.; Xue, H.; Hongwei, J.; Zhengong, Y.; Zhanguo, Z.; Jinzhu, Z.; Rongsheng, Z. Identification and validation of major QTLs and epistatic interactions for seed oil content in soybeans under multiple environments based on a high-density map. *Euphytica* **2017**, *213*, 1-14.
27. Qiu, B.X.; Arelli, P.R.; Sleper, D.A. RFLP markers associated with soybean cyst nematode resistance and seed composition in a 'Peking'×'Essex'population. *Theoretical and Applied Genetics* **1999**, *98*, 356-364.
28. Reinprecht, Y.; Poysa, V.W.; Yu, K.; Rajcan, I.; Ablett, G.R.; Pauls, K.P. Seed and agronomic QTL in low linolenic acid, lipoxygenase-free soybean (Glycine max (L.) Merrill) germplasm. *Genome* **2006**, *49*, 1510-1527.
29. Seo, J.H.; Kim, K.S.; Ko, J.M.; Choi, M.S.; Kang, B.K.; Kwon, S.W.; Jun, T.H. Quantitative trait locus analysis for soybean (Glycine max) seed protein and oil concentrations using selected breeding populations. *Plant Breeding* **2019**, *138*, 95-104.
30. Shibata, M.; Takayama, K.; Ujiie, A.; Yamada, T.; Abe, J.; Kitamura, K. Genetic relationship between lipid content and linolenic acid concentration in soybean seeds. *Breeding science* **2008**, *58*, 361-366.
31. Smallwood, C.J.; Gillman, J.D.; Saxton, A.M.; Bhandari, H.S.; Wadl, P.A.; Fallen, B.D.; Hyten, D.L.; Song, Q.; Pantalone, V.R. Identifying and exploring significant genomic regions associated with soybean yield, seed fatty acids, protein and oil. *Journal of crop science and biotechnology* **2017**, *20*, 243-253.
32. Tajuddin, T.; Watanabe, S.; Yamanaka, N.; Harada, K. Analysis of quantitative trait loci for protein and lipid contents in soybean seeds using recombinant inbred lines. *Breeding science* **2003**, *53*, 133-140.
33. Wang, X.; Jiang, G.-L.; Green, M.; Scott, R.A.; Hyten, D.L.; Cregan, P.B. Quantitative trait locus analysis of saturated fatty acids in a population of recombinant inbred lines of soybean. *Molecular Breeding* **2012**, *30*, 1163-1179.
34. Wang, X.; Jiang, G.-L.; Green, M.; Scott, R.A.; Song, Q.; Hyten, D.L.; Cregan, P.B. Identification and validation of quantitative trait loci for seed yield, oil and protein contents in two recombinant inbred line populations of soybean. *Molecular Genetics and Genomics* **2014**, *289*, 935-949.
35. Wang, J.; Chen, P.; Wang, D.; Shannon, G.; Zeng, A.; Orazaly, M.; Wu, C. Identification and mapping of stable QTL for protein content in soybean seeds. *Molecular Breeding* **2015**, *35*, 1-10.
36. Li, X.; Shao, Z.; Tian, R.; Zhang, H.; Du, H.; Kong, Y.; Li, W.; Zhang, C. Mining QTLs and candidate genes for seed protein and oil contents across multiple environments and backgrounds in soybean. *Molecular Breeding* **2019**, *39*, 1-16.
37. Zhang, W.K.; Wang, Y.J.; Luo, G.Z.; Zhang, J.S.; He, C.Y.; Wu, X.L.; Gai, J.Y.; Chen, S.Y. QTL mapping of ten agronomic traits on the soybean (Glycine max L. Merr.) genetic map and their association with EST markers. *Theoretical and Applied Genetics* **2004**, *108*, 1131-1139.

38. Leite, D.C.; Pinheiro, J.B.; Campos, J.B.; Di Mauro, A.O.; Unêda-Trevisoli, S.H. QTL mapping of soybean oil content for marker-assisted selection in plant breeding program. *Genet. Mol. Res* **2016**, *15*, 10-4238.
